# The Effect of Differing Levels of Intrasexual and Intersexual Selection on Survival and Reproduction Under a Heatwave

**DOI:** 10.1002/ece3.72778

**Published:** 2026-02-01

**Authors:** Karendeep K. Sidhu, Paul Caplat, Greta Bocedi, Natalie Pilakouta, Lesley Lancaster

**Affiliations:** ^1^ University of Aberdeen Aberdeen Scotland; ^2^ Queens University Belfast Belfast Northern Ireland; ^3^ University of St Andrews St Andrews Scotland

**Keywords:** heatwaves, mating system, reproductive success, sexual selection, survival

## Abstract

Heatwaves are set to become more common due to climate change, and the potential of heatwaves to damage a species' populations is becoming more apparent. One way heatwaves can affect reproduction is by changing the dynamics of precopulatory mating behaviours, which can detrimentally impact individuals' survival and reproductive success. However, little work has been done to investigate how different levels of intrasexual and intersexual selection in precopulatory behaviours, such as male–male competition or female‐mate choice, modulate fitness responses to heatwave. Here we investigate how differing levels of male competition and female choice impact survival and reproductive success when a heatwave event occurs, using cool‐adapted Scottish populations of the burying beetle *Nicrophorus vespilloides* as a model system. We implemented three treatments for precopulatory conditions: monogamy (one male, one female, no male–male competition), polyandry with male competition (one female, two males, where males and females could freely interact), and polyandry with low male competition (one female, two males, where males could not interact with each other or the female freely). We then subjected the beetles to a heatwave event (3 days at 25°C) at the time of mating. We found that reproductive fitness decreased under a heatwave in the polyandry/low male competition treatment, compared to both the monogamy and the male competition treatments. Our results indicate that differing levels of intrasexual and intersexual selection can moderate the detrimental effects of heatwaves in wild species.

## Graphical abstract

Precopulatory mating behaviour, are important for fitness and can be disrupted by heatwave's. Here we assess how differing levels of intrasexual and intrasexual selection in precopulatory mating behaviours impacts fitness responses to heatwaves. We find that the likelihood of having a brood is impacted by differences in intrasexual and intersexual selection in response to a heatwave.

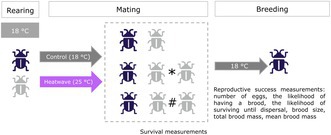

## 1 Introduction

Heatwaves are becoming more common, intense, and longer due to climate change (Meehl and Tebaldi 2004; Diffenbaugh et al. 2007; Déqué 2007; Perkins et al. 2012; Chirault et al. 2015; Mazdiyasni and AghaKouchak 2015). Heatwaves are extreme climatic events, defined as periods when daily maximum temperatures are, on average, 5°C higher than the average daily maximum for more than 5 days (Frich et al. 2002). They have been shown to impact both survival (Bauerfeind and Fischer 2014; Blanckenhorn et al. 2014; Porcelli et al. 2017; Rukke et al. 2018; Chevrier et al. 2019; Wild et al. 2019; Sales et al. 2021) and reproduction (Porcelli et al. 2017; Rukke et al. 2018; Sales et al. 2018; Green et al. 2019; Ratz et al. 2024), potentially leading to sterility in a variety of animal species (Nguyen et al. 2013; Chevrier et al. 2019). The decrease in reproductive success caused by heatwaves can lead to a decrease of population size in future generations and, in turn, a decrease in genetic diversity potentially leading to further population decline.

Sexual selection, that is selection that arises from fitness differences associated with nonrandom success in the competition for fertilisation (Shuker and Kvarnemo 2021), has the potential to impact the effect of heatwaves on reproductive success (Berger et al. 2014; García‐Roa et al. 2020). For example, there is increasing evidence that manipulating operational sex ratios (OSR) in the laboratory, and thus the strength of sexual selection, such as through enforced polyandry or monogamy, can impact reproduction and survival outcomes after a heatwave (Vasudeva et al. 2021; Moiron et al. 2022). Polyandry occurs when each female mates with multiple males within a single reproductive bout and can impact sexual selection in a multitude of ways (Kvarnemo and Simmons 2013), as well as potentially influencing female reproductive success under thermal stress. For example, polyandry allows females to improve their fertility when males' reproductive function is compromised after a heatwave (Vasudeva et al. 2021; Moiron et al. 2022). Polyandry can also weaken precopulatory sexual selection in males by reducing a male's ability to monopolise females, which can lead to stronger postcopulatory sexual selection through postcopulatory cryptic female choice and sperm competition (reviewed in Kvarnemo and Simmons (2013)).

The consequences of polyandry may be particularly important in mitigating the potential negative fitness effects of heatwave exposure. Polyandry could lead to an increase in resources through nuptial gifts or ejaculation and an increased fertility assurance of sperm (Birkhead and Pizzari 2002; Uller and Olsson 2005) for example, to counter the impacts of male infertility on female fertility (Wetton and Parkin 1997; Vasudeva et al. 2021). Fertility assurance is particularly important as the thermal stress imposed by heatwaves can result in decreasing sperm mobility (Porcelli et al. 2017), sperm counts (Nguyen et al. 2013; Chevrier et al. 2019), sperm viability (Martinet et al. 2020) and sperm competitiveness (Sales et al. 2018), which decreases the ability of males to reproduce (Nguyen et al. 2013). This can lead to males siring fewer offspring (Sales et al. 2024), and in some cases to male sterility (Sales et al. 2018). Polyandry can then act as a mechanism to ensure female fertility through acquisition of sperm from multiple males thus hedging against increased male infertility following heatwaves (e.g., Sutter et al. (2019); Vasudeva et al. (2021) and Moiron et al. (2022)). Further, polyandry can improve individual and population survival, relative to monogamy, at elevated temperatures (see Holland (2002), Omkar and Pandey (2010), Grazer and Martin (2012), Plesnar‐Bielak et al. (2012) and Parrett and Knell (2018)). Polyandry also can maintain higher genetic diversity in the population by reducing inbreeding. In the long term, the potentially higher genetic diversity in polyandrous populations could help the population persist in novel, fluctuating (Candolin and Wong 2015; Lumley et al. 2015) or extreme environmental conditions such as heatwaves.

The potential fitness benefit of polyandry under thermal stress could however be diminished by its negative consequences, such as increased harm associated with competitive behaviours through increased intrasexual selection and mating activities for both males and females (Kvarnemo and Simmons 2013). Polyandry can increase sexual conflict, as when males and females have unequal access to mates, there is an increased mismatch between the optimal mating strategies for fitness between the sexes (Holman and Kokko 2013; Rowe and Rundle 2021). For example, increased harassment can cause a reduction in females' longevity (Martin and Hosken 2003), as well as a reduction in female choice and consequent intersexual selection, and more physical harm (Burke and Holwell 2023) through increased mating and competitive interactions, all of which can potentially result in reduced survival for males and females.

However, there is still a gap in our understanding of how sexual selection and polyandry may affect reproductive success when a heatwave occurs during mating, as opposed to when the heatwave is experienced either before (Vasudeva et al. 2021; Moiron et al. 2022) or after mating has occurred (Pilakouta et al. 2023). Heatwaves at different stages of reproduction have been shown to have a varied impact, with heatwaves occurring closer to the point of mating being the most detrimental to reproductive success (Pilakouta et al. 2023). Therefore, polyandry may provide different benefits and costs to an individual's reproductive success depending on when the heatwave occurs. For instance, heatwaves experienced during mating could exacerbate the direct, energetic or injury risk costs of engaging in polyandry. Additionally, the potential benefits and costs of polyandry may be modulated by the environment (Candolin and Wong 2015), such that we still do not know the net fitness effect of polyandry in thermally challenging environments. Specifically, little is known about how heatwave‐related fitness effects are impacted by differing levels of male–male competition (intrasexual selection) and male harassment, or female choice (intersexual selection) (Pilakouta and Ålund 2021), under monogamy and polyandry, which is a gap this study aims to address.

In this study, we investigate how differing levels of intersexual and intrasexual selection imposed by the presence or absence of precopulatory female choice and male–male competition impact reproductive fitness when there is a heatwave at the point of mating. We also asked whether adult survival after the heatwaves has any further impact on differences in reproductive success between different levels of intrasexual and intersexual selection. To address these questions, we use a cool‐adapted Scottish population of burying beetle *Nicrophorus vespilloides* , a species where females can mate with multiple males and store sperm until they have access to a small vertebrate carcass for breeding. They display facultative biparental care, where mating and parental care behaviours coevolved because of sexual conflict over mating and paternity assurance of broods (Royle and Hopwood 2017). The intensity of sexual selection in this species has been shown to be sensitive to operational sex ratios, the availability of resources and the relative competitive ability of individuals (reviewed in Royle and Hopwood (2017)). They have also shown the ability to respond to temperature changes through rapid changes in mating behaviour (Quinby et al. 2020), and parental care behaviour (Grew et al. 2019; Pilakouta et al. 2023; Sidhu et al. 2024).

We subjected the beetles to three treatments to impose differing levels of intrasexual and intersexual selection: (1) monogamy (no intrasexual selection and no intersexual selection), (2) polyandry with precopulatory male competition (more intrasexual competition, limited intersexual selection), and (3) polyandry with low or no precopulatory male competition (restricted intrasexual competition, but retained intersexual selection through female choice). We first compare treatments 1 and 2 to assess if monogamy and polyandry differ in reproductive success in response to a heatwave occurring during mating. We then compare treatment 2 to treatment 3 to assess fitness impacts of intrasexual selection occurring under polyandrous matings during a heatwave. Though our polyandry treatments differ in both levels of female mate choice (intersexual selection; as females are not subject to male harassment in the second treatment) and male–male competition (intrasexual selection) for clarity, we identify them through differences in male competition throughout. Our treatments differ in their costs and benefits which we predict will impact behavioural and fitness responses to heatwave when mating is occurring. Monogamy (treatment 1) obviously provides no benefit from multiple mating, but also, there is no potential harm from harassment. Polyandry with male competition (treatment 2) provides potential benefits of female mating multiply but also increased harm with increased direct male–male competition (increased intrasexual selection). Such harms could be incurred by females, as increased harassment and coercive mating could decrease female condition and/or survival, or by males, potentially leading to effective monogamy due to male death after exertion or injury of competition during a heatwave. Alternatively, polyandry with low male competition (achieved by limiting males' interactions with each other and the female; treatment 3) provides the benefits of female multiple matings (e.g., access to more genetically diverse or functional sperm) and limits its cost by preventing male harassment and potential harm to males coming from direct competition. This treatment also increases the opportunity for female choice, as there is less opportunity for male coercive matings.

We predict that overall, polyandry will increase reproductive success over monogamy via bet‐hedging for fertility assurance (Yasui and Garcia‐Gonzalez 2016; Yasui and Yamamoto 2021) and more so under heatwaves due to impact of high temperature on male fertility. We also predict that treatment 3 (limited male competition) will increase fitness over unrestricted polyandry (treatment 2). Again, this effect may be magnified under heat stress, when agosistic interactions of competition and harassment are expected to be more costly. Therefore, under nonheatwave conditions, polyandry should result in greater fitness than monogamy (treatment 2 vs. treatment 1), and this difference should be greater under heatwave conditions. Additionally, fitness differences among polyandrous scenarios with differing male competition regies (2 vs. 3) should also be greater under heatwave conditions than under ambient temperatures. This is because the positive fitness effect of polyandry compared to monogamy under thermal stress could be weakened if male harassment increases and/or its effects on female survival are magnified under a heatwave.

## 2 Methods

### 2.1 Study Species

Burying beetles, *Nicrophorus vespilloides*, breed on small vertebrate carcasses, displaying facultative biparental care at both preand posthatching offspring stages (Scott 1998; Royle and Hopwood 2017). The prehatching care consists of removing feathers or fur from the carcass and applying antimicrobial secretions to reduce bacterial and fungal growth, and lay eggs in the surrounding soil (Scott 1998; Royle and Hopwood 2017). When the eggs hatch, parents provide posthatching care, which includes feeding and grooming of larvae, and maintaining or guarding the carcass. Under laboratory conditions at 18°C, the larvae disperse from the carcass about 7 to 10 days after hatching, pupate and eclose (i.e., emerge from pupae) as adults about 18 to 24 days after dispersal. They become sexually mature about 2 weeks after eclosion (Scott 1998; Royle and Hopwood 2017). To find mates in the wild, males will release species‐specific pheromones to attract females and will mate regardless of whether there is a carcass present (Royle and Hopwood 2017). Therefore, most females are premated when a carcass becomes available, and most broods have mixed paternity (Royle and Hopwood 2017). To attain a carcass, which is essential for reproduction, there is intraspecific competition as well as interpair fights until the carcass has one dominating pair. However, if the carcass is big enough, multiple pairs can breed on one carcass (Royle and Hopwood 2017).

### 2.2 Experimental Population

The population used in this study consisted of third‐generation beetles, originally collected from Kincorth Hill Local Nature Reserve (57°6′ 55.74″ N, 2°6′ 11.89″ W), Tollohill wood (57°6′ 40.61″ N, 2°7′ 54.89″ W) in Aberdeen and Den Wood (57°22′ 0.79″ N, 2°19′ 12.19″ W) in Inverurie, UK, between June and September 2022. To avoid inbreeding, mated individuals had no common ancestors at any point during two generations of laboratory rearing before the study. Beetles were housed individually in transparent plastic containers (124 × 82 × 22 mm), half‐filled with moist soil and kept at 18°C, with a 12‐h:12‐h light/dark cycle. We fed the beetles small cubes of organic beef twice a week. At the start of the experiment, we selected F2 generation females and mated them to unrelated males, which resulted in *N* = 56 families used for this experiment. The offspring of these mated pairs, F3, became our experimental population.

We split the clutches (F3 generation) of each of our 56 families equally between the mating and heatwave treatments, just before the mating trials began, so that we could match the number of families equally for each treatment. This resulted in the following mated pairs: monogamy control (*N* = 27), polyandry, with male competition control (*N* = 23), polyandry with low male competition control (*N* = 30), monogamy heatwave (*N* = 29), polyandry with male competition heatwave (*N* = 26), polyandry with low male competition heatwave (*N* = 22). Some beetles were not mated either due to age, when they were over 10–15 days post eclosion, or due to uneven sex ratios.

### 2.3 Mating Treatment and Adult Survival

**FIGURE 1:**
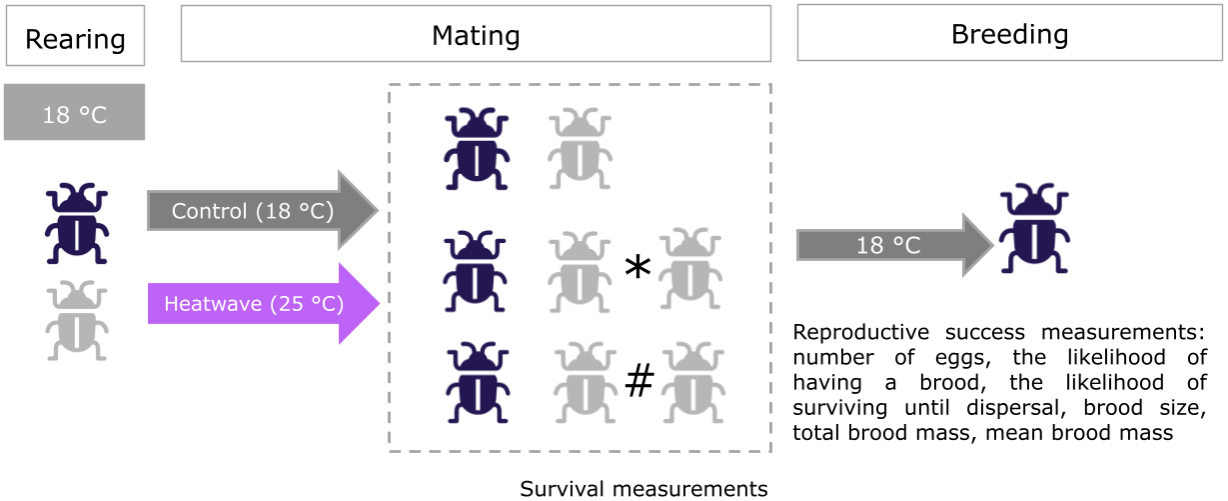
Experimental design showing when the temperature treatments (heatwave or control) took place (at the mating stage). The heatwave temperature was 25°C and the control 18°C, both of which lasted for 3 days, Blue = Female and Grey = Male. The mating treatments consisted of monogamy (one male, one female), polyandry with low male competition (*) and polyandry with male competition (#). After the temperature treatment, the mating groups had their survival recorded. The males were then removed and placed at −20°C, and females from all treatments were provided with a carcass for breeding at 18°C. We then measured the reproductive success: Egg number, brood size, mean larval mass, total brood mass, the likelihood of having a brood, and the likelihood of dispersal.

**FIGURE 2:**
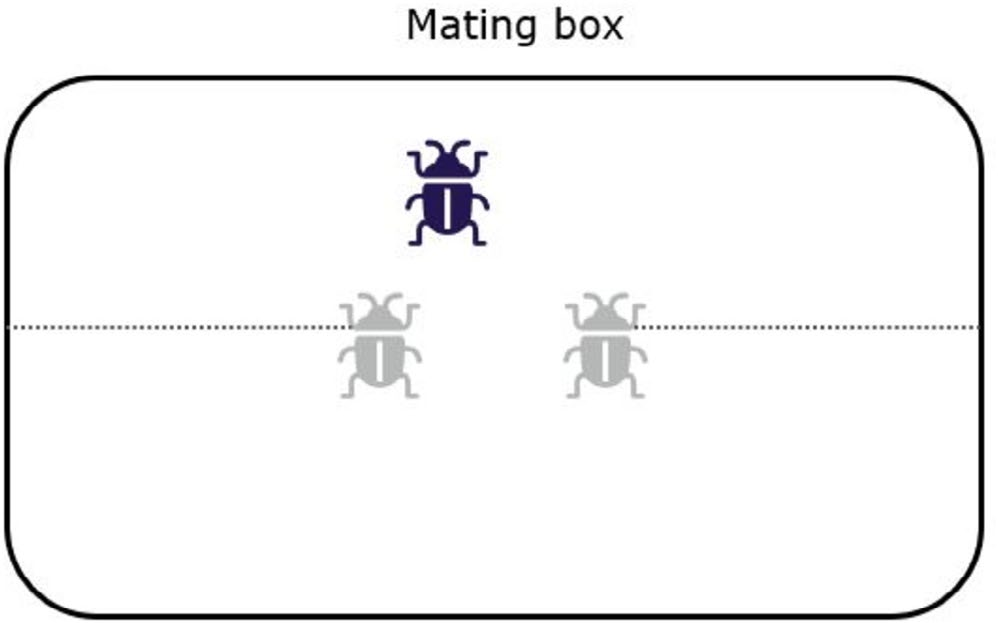
The layout of the mating boxes in the polyandry no male competition treatment. Males (grey) were placed on leashes where they could move in only half the breeding box and could not physically interact with the other males, nor harass the female; whereas females were free to roam the box.

We used a 2 × 3 factorial design with three precopulatory competition treatments crossed with a heatwave and control treatment (experimental design outlined in Figure 1). In the monogamy treatment, there was one male and one female. In the two polyandry treatments, “with precopulatory male competition”, treatment 2, and “low precopulatory male competition”, treatment 3, there was one female and two males. Low male competition was ensured by the males being secured to opposite sides of the mating arena such that they could not physically interact (Figure 2). Though in our low male competition treatment, males could still compete through visual (body size differences) or olfactory cues (pheromones) (Royle and Hopwood 2017), as well as through how actively they approached the female.

For all mating treatments, we fitted all males with a collar made from unscented and unwaxed dental floss (Oral B Essential Floss Unwaxed), which was placed around their neck and had a long tail, which we used as a leash. After the collar was fitted on the males, we ensured they had freedom of movement and allowed a 24‐h acclimatisation period where they were housed individually under standard laboratory conditions. For the polyandry treatments, one of the males had a small dot placed on the collar with a pen to distinguish between the two males, the male with the dot being denoted as male one and the other as male two. After the acclimatisation period, the leashes were either cut to a shorter length to allow freedom of movement or kept long, depending on the treatment. For the polyandry with competition treatment (2) and the monogamy treatment (1), the collar was still attached to each male, but with the tether cut short and not attached to the box, while for the polyandry with low male competition treatment (3), a longer leash was kept and was attached using masking tape to the mating boxes. In the polyandry with low male competition treatment, each male had roaming room that was just under half of the box to ensure they had freedom of movement and could freely interact with the females while also ensuring that the males could not interact with one another, as they were attached to opposite ends of the breeding boxes (Figure 2).

The mating pairs were placed in mating boxes (174 × 115 × 60 mm) with a 1‐cm layer of moist soil. The mating boxes were then placed into a temperature‐controlled incubator (LMS Series 1A Model 280NP) for 3 days with a 12‐h:12‐h light/dark cycle, at 40% ± 10% humidity within the incubator, which was maintained to match lab humidity conditions, using magnesium chloride hexahydrate 98% salt mixed with water until saturation. Fresh saturated solution was made once the humidity began to drop below the desired level. We monitored humidity levels using Fisherbrand Traceable Jumbo Thermo‐Humidity Metres. The incubator was either set at 18°C, which is the same as standard laboratory conditions, or to a three‐day heatwave at 25°C (air temperature). A heatwave of 25°C for 3 days was selected as it represents potential conditions that burying beetle populations might experience in Scotland where the ancestral population was collected (Ready Scotland 2022). This temperature also represents the upper limit of heatwaves Aberdeenshire experienced in recent years (Met Office 2018), making our simulated heatwave ecologically relevant.

After the three‐day mating period, the mating boxes were removed from the incubator and placed at standard laboratory conditions of 18°C where their survival was recorded. Following the mating treatment, we removed any surviving males, measured their pronotum and euthanized them at −20°C. As the males had their pronotum measured after mating to not further stress the males and due to time constraints, we could not measure the pronotum of some males that did not survive mating, as their bodies were not found or were partially eaten. Due to this, male pronotum measurements were not included in our analysis of male survival, although possible effects are potentially limited as we chose males of similar size for the experiment. The surviving females were placed in breeding boxes (174 × 115 × 60 mm) with a 1‐cm layer of moist soil alongside a freshly thawed mouse carcass (Livefoods Direct Ltd) of a standardised size (22.5–26 g). In this limited amount of soil, the number of eggs laid was easily visible at the bottom of the transparent box (Monteiro and Lyons 2012).

### 2.4 Breeding and Reproductive Success

Female beetles were weighed before mating to reduce handling before they were placed into the breeding boxes, and after their own larvae had dispersed away from the carcass. In the case of pairings which resulted in no eggs, the beetles were still weighed when the carcass began to show significant fungal growth, usually about 4–5 days after providing the carcass. We recorded the number of eggs laid 48 h after mating and took a further two recordings at 10 am and 4 pm after the initial recording. We used the recording with the highest number of eggs for further analysis. Broods were then left to complete their development and monitored for dispersal twice daily. Dispersal was determined when all larvae in the brood had moved from the carcass into the surrounding soil, which happened 5–10 days after hatching. To determine the reproductive success of each pair where the female survived (monogamy control (*N* = 22), polyandry with male competition control (*N* = 22), polyandry with low male competition control (*N* = 27), monogamy heatwave (*N* = 19), polyandry with male competition heatwave (*N* = 18), polyandry with low male competition heatwave (*N* = 16)), we recorded the number of eggs and the number of dispersing larvae (brood size), and calculated the likelihood of having a brood (likelihood of the brood size being greater than zero). We also measured the total brood mass by weighing the whole brood after all the larvae had dispersed away from the carcass. Total brood mass was then divided by the number of larvae, providing an estimate of the mean larval mass. We also calculated the likelihood of survival to dispersal, which is the number of offspring that survived to the dispersal stage, using the initial number of eggs laid and brood size at dispersal, although the reduction in the brood size from the egg number could be due to brood culling, infertility or differences in survival due to the temperature treatments.

### 2.5 Statistical Analysis

All data were analysed using R version 4.0.2 (R core Team 2020). GLMMs and LMMs were performed using the functions “glmer” and “lmer” in the R package “lme4” (Bates et al. 2015). All models are detailed in Table S1.

We first tested if heatwaves impacted monogamy or polyandry reproductive success differently. We ran models with monogamy and polyandry (with competition) and their interaction with heatwaves, as well as female mass before mating and carcass mass as fixed effects. Female mass before mating and carcass mass were included as these can impact reproductive success measurements. For random effects, we included the female's family ID and the male one's family ID as random effects. Male two's family ID could not be used as a random effect as two males were present only in the polyandry treatments. We then compared models with different distributions (gaussian, possion, and negative binomial) to assess which distribution best fit the distribution of our data. We also compared models with and without random effects for each of our reproductive success measurements: number of eggs, brood size, likelihood of having a brood, likelihood of survival until dispersal has GLMM and GLM compared and mean larval mass and total brood mass had LM and LMMS compared, we then chose the model with the lower AIC was chosen; if the models differed in their AIC by < 2, the model without random effects was chosen. Which resulted in the number of eggs analysed with GLMs with negative binomial distribution and brood size analysed with GLMM with negative binomial distribution. The likelihood of dispersal and the likelihood of having a brood were analysed using GLMs with the binomial distribution. The mean larval mass and total brood mass were normally distributed and therefore analysed with LMMS.

We then tested if heatwaves impacted reproductive success differently when there were differing levels of competition in polyandry. We ran models with our two polyandry treatments (with competition, and with low competition) and their interaction with the heatwave and female mass before mating and carcass mass as fixed effects, random effects included male one family ID and female family ID. These models underwent the same model selection outlined above, resulting in the number of eggs and brood size analysed with GLMs with negative binomial distribution. The likelihood of dispersal and the likelihood of having a brood were analysed using GLMs with the binomial distribution. The mean larval mass and total brood mass were normally distributed and, therefore, analysed with LMMS.

To test if the heatwaves, monogamy or polyandry impacted male or female survival, we analysed female survival using GLMMs with binomial distributions. We included female mass before mating, and either monogamy and polyandry, or the differing level of male competition within polyandry, in interaction with a heatwave, as fixed predictors, and female's family ID as a random effect. We tested male survival with a similar GLMM but included mating ID (to account for males in the same mating box) and male family ID as random effects.

We also tested whether male survival impacted reproductive success, as male death could mean lower fertilisation opportunities for females or less harm and/or increased resources through the consumption of the dead male. We first tested if a male dying in the monogamy treatment impacted reproductive success. In these models, we included the effect of heatwave, male survival and their interaction on the number of eggs and brood size (GLMM, negative binomial distribution), on the likelihood of dispersal and of having a brood (GLMM, binomial distribution), and on mean larval mass and total brood mass (LMMs). All the models for reproductive success included carcass mass and female mass before mating as fixed effects. We then tested whether heatwave, male survival and their interaction affected reproductive success in the two polyandry treatments. We created a new variable for the three different levels for male survival (no males survived, one male survived and both males survived) and tested all reproductive success measurements. Our models included interaction between heatwaves and different levels of survival in polyandry as fixed effects, and female and male family ID as random effects with the number of eggs and brood size (GLMM, negative binomial distribution), on the likelihood of dispersal and of having a brood (GLMM, binomial distribution), and on mean larval mass and total brood mass (LMMs).

## 3 Results

### 3.1 Effect of Heatwave and Precopulatory Sexual Selection on Adult Survival

#### 3.1.1 Monogamy vs. Polyandry

Female survival was negatively impacted by the heatwave (*z* = −1.90, *p* = 0.02; Table S2) but was unaffected by mating treatment or by its interaction with the heatwave (Table S2). Male survival was also negatively impacted by the heatwave (*z* = −2.32, *p* = 0.004), but not by the mating treatment or by their interaction (Table S2).

#### 3.1.2 Differing Levels of Male Competition Within Polyandry

Female survival was not impacted by the heatwave when considered just within the polyandry treatments, nor by the interaction between heatwave and differing levels of male competition (Table S2). Male survival was not impacted by the heatwave or level of male competition when considered only within polyandry treatments, nor by the interaction between these, each of which was marginally nonsignificant (heatwave: *z* = −0.93, *p* = 0.06; interaction: *z* = −1.27, *p* = 0.07; Table S2).

### 3.2 Effect of Heatwave and Precopulatory Sexual Selection on Reproductive Success

#### 3.2.1 Monogamy vs. Polyandry

**FIGURE 3:**
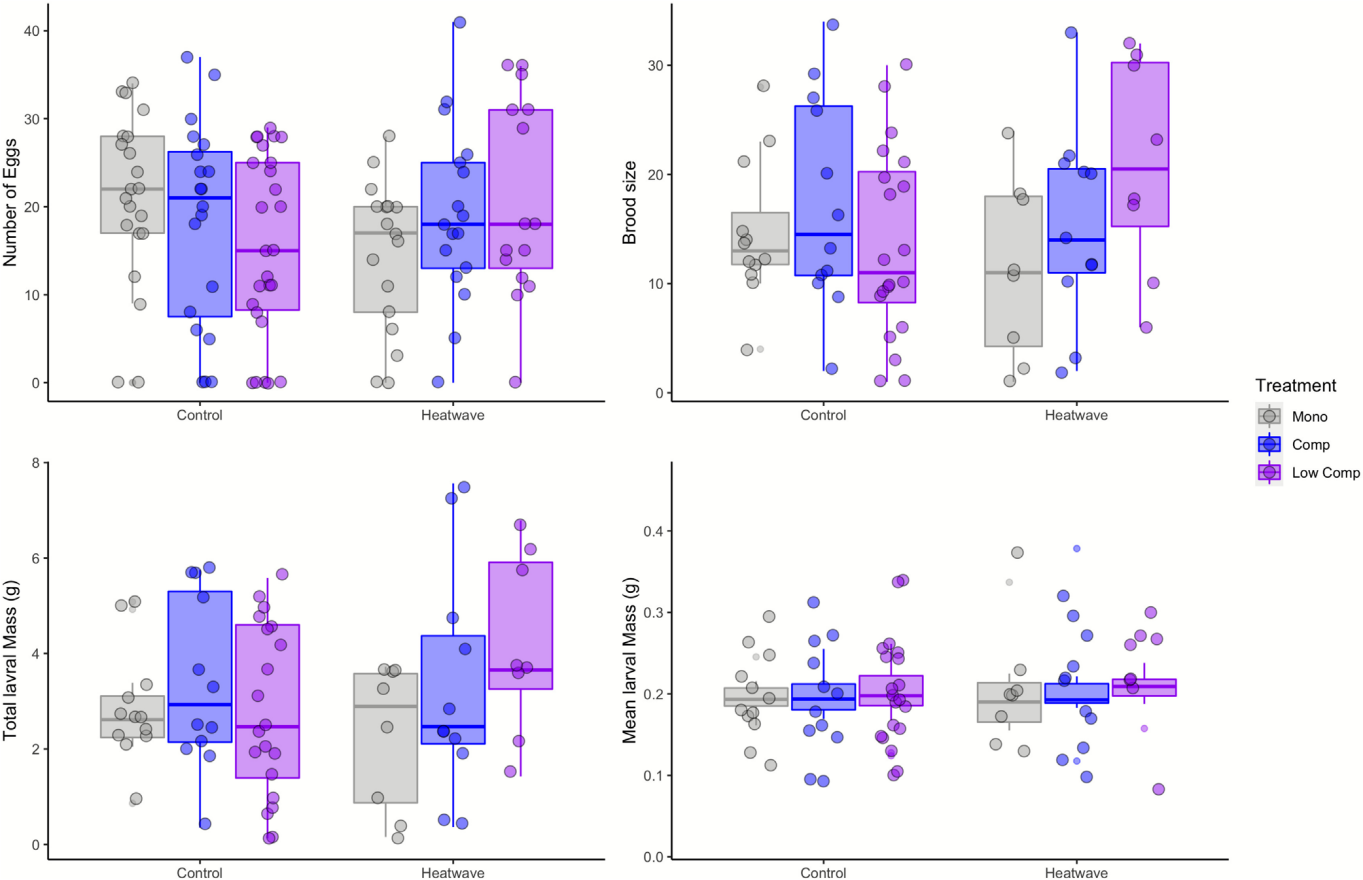
The effect of heatwave exposure on (a) the number of eggs between our mongamy (grey), male compeitition(purple) and low competition male (pink) treatments (b) brood size between our mongamy (grey), male compeitition(purple) and low competition male (c) total larval mass between our mongamy (grey), male compeitition (purple) and low competition male (d) mean larval mass between our mongamy (grey), male compeitition (purple) and low competition male The horizontal bar in the boxes represents the median, the lower and upper hinges of the box correspond to the first and third quartiles and the lower and upper whiskers extend from the hinge to the smallest and largest value no further than 1.5 × the interquartile range from the hinge. Each data point (circles) represents one mating.

None of the reproductive success measurements (i.e., number of eggs, likelihood of surviving until dispersal, the likelihood of having a brood, brood size, total larval mass and mean larval mass) were significantly impacted by monogamy or polyandry treatments, the heatwave treatment, or the interaction between these factors (Figure 3; Table S3).

#### 3.2.2 Differing Levels of Male Competition Within Polyandry

**FIGURE 4:**
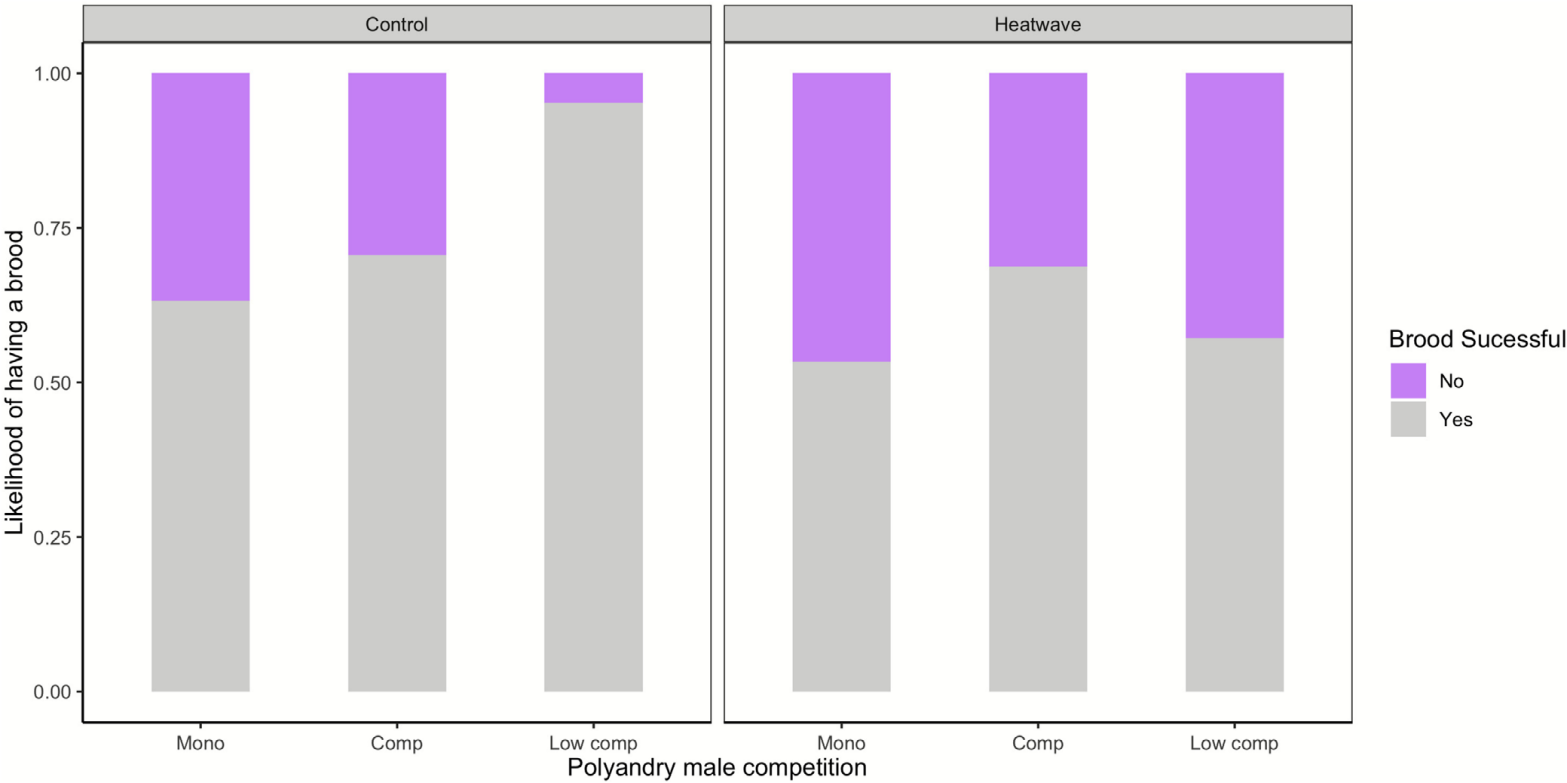
Effect of heatwave exposure on the likelihood of having a brood (at least one offspring at the end of the breeding period), between male competition vs. no competition treatments, both under polyandry.

The differing levels of male competition, heatwave, or interaction between these two groups did not significantly impact most of the reproductive success measurements when considered only within polyandry treatments (Figure 3; Table S4). However, the likelihood of having a brood was affected by the level of male competition (*z* = 2.02, *p* = 0.04; Figure 4), increasing in the low male competition treatment, as well as by the interaction between level of male competition and heatwave, with a higher likelihood of having a brood in the low male competition treatment (low male harassment, low male–male competition) under a heatwave (*z* = −2.01, *p* = 0.04; Table S4 (Table S3)). The opposite trend is observed in the control treatment (Figure 4).

As the heatwave impacted male survival (Table S2), we tested if changes in male survival influenced reproductive success in any of our treatments. There was no effect of male survival on reproductive success in any of our treatments: monogamy (Table S5) or polyandry (Table S6).

We also conducted a power analysis using the pwrss package (Bulus and Jentschke 2025), which indicated that given a normal distributed (as with total and mean brood mass) we would need *N* = 474, in order to obtain 80% power to detect an effect of *β*_1_ = −0.10 at *α* = 0.05. For our nonnormally distributed data (number of eggs and brood size) we conducted a power analysis assuming poisson distribution which indicated we would need *N* = 562 to provide 80% power to detect an effect of *β*_1_ = −0.10 at *α* = 0.05 to detect significant differences in our treatments (temperature and level of male–male competition) effects on our response variables if such true differences were present (Table S6). This result reinforces our reported negative finding, as any true effect requiring such high sample sizes to be detected is unlikely to be ecologically meaningful within standard population densities of burying beetles in the wild.

## 4 Discussion

Our study tested the effect of heatwaves during mating and differing levels of intersexual and intrasexual selection on reproductive success and survival, by having different levels of precopulatory female mate choice and male–male competition: monogamy, polyandry with male competition and polyandry with low male competition. We found that the amount of male competition impacted reproductive success in a heatwave, although this effect was not in the direction we predicted, as the likelihood of having a brood decreased in the polyandry with low male competition compared to the polyandry with male competition treatment when there was a heatwave. This effect was not mediated by differences in male survival, as there were no differences in male survival between these two treatments (Table S2). Although we expected polyandry to increase multiple dimensions of fitness under nonheatwave conditions and potentially also under thermal stress, there were no differences in other reproductive success measurements (number of eggs, brood size and total brood mass, larvae mean mass and likelihood of survival until dispersal) between monogamy, polyandry or heatwave treatments, or their interactions. Although we found an effect of heatwaves on male and female survival, this effect did not interact with mating treatment, and we did not see an impact of male survival on reproductive success.

The result that the potential increase in female choice, together with polyandry with low male–male precopulatory competition and male harassment, decreases the likelihood of having a brood when mating during a heatwave, was unexpected, as these conditions should provide the most benefits (e.g., fertility assurance) and the least harm to females. Though our results are in line with other studies that find the likelihood of having a brood to be negatively impacted by a simulated heatwave (Quinby et al. 2020; Pilakouta et al. 2023), as well as differing levels of intrasexual and intersexual selection (Omkar and Pandey (2010)), our study is the first to test the combination of these factors. Our results may indicate that when females had more freedom of choice and were less harassed, they engaged less with mating during thermal stress, thus resulting in lower reproductive success. Females choosing not to engage in breeding has been reported when ambient temperatures increase (Quinby et al. 2020), though not previously in heatwaves. This highlights a potential difference in the effect of heatwaves on reproductive decisions depending on when heatwaves happen in the mating and breeding cycle. Previous studies showed that females use polyandry to increase fertility after a heatwave has occurred (Vasudeva et al. 2021; Moiron et al. 2022), though in these studies only the male was exposed to a heatwave. Our findings suggest that this is not the case when the heatwave happens as mating is occurring, and instead females may forgo mating opportunities potentially because of thermal stress. The reduction in the likelihood of having a brood during a heatwave could be further attributed to the combined effects of reduced female engagement with mating and potential alterations to male sperm by temperature changes (Katsuki and Miyatake 2009; Nguyen et al. 2013). This may happen through a variety of mechanisms, including: decrease in male sperm count (Katsuki and Miyatake 2009; Nguyen et al. 2013), decrease in the duration of sperm transfer to females at higher temperatures (Katsuki and Miyatake 2009), alterations in the viscosity of seminal fluids (Purchase et al. 2010) or seminal proteins (Martinet et al. 2023). The combination of these effects may explain why we see a decrease in the likelihood of having a brood only in the heatwave polyandry with low male competition treatment.

It is surprising that heatwave and mating treatments did not affect any of all the other reproductive success measurements (number of eggs, brood size and total brood mass, larvae mean mass and likelihood of dispersal). Previous studies have found a negative impact of heatwave on egg‐to‐adult viability in *Drosophila* (Porcelli et al. 2017; Green et al. 2019); as well as a decrease in offspring lifespan in red flour beetles ( *Tribolium castaneum* ) (Sales et al. 2018), and brood size reductions in burying beetles (Pilakouta et al. 2023) and in field crickets (*Gryllus bimaculatus*) (Ratz et al. 2024) as well as smaller offspring size in burying beetles (Pilakouta et al. 2023). The positive effects of polyandry after a heatwave have also been reported on likelihood of having a brood in *Drosophila* (Sutter et al. 2019) and number of offspring in the flour beetle ( *Tribolium castaneum* ) (Vasudeva et al. 2021; Moiron et al. 2022). Additionally, differing levels of precopulatory competition and mate choice have also been shown to impact on egg viability and number of eggs (Omkar and Pandey 2010). It is also surprising that polyandry did not improve fitness in the nonheatwave treatment and, even more so, under heatwave conditions. This may be for a few reasons. We may not have seen a difference in number of eggs as females do lay unfertilised eggs (Eggert et al. 1998; Lambert and Smiseth 2022), so any impact of either heatwave or level of male competition may be obscured, though this is unlikely as brood size was also not impacted. That brood size was not affected by our treatments may be due to sperm not being compromised due to the timing of the heatwave (Sales et al. 2021), the duration of exposure or spermatogenesis not being impacted by the heatwave, though this is difficult to assess as there is limited work on the impact of heatwaves on sperm viability within *Nicrophorus vespilloides* . Sperm not being compromised by the heatwave may explain why we did not see a positive impact of polyandry on brood size as the potential benefit of sperm assurance (Birkhead and Pizzari 2002; Uller and Olsson 2005; Kvarnemo and Simmons 2013) would not accrue. Additionally, the majority of the heatwaves outlined in the studies above had 5‐day heatwaves as opposed to 3‐day heatwaves, which we chose as our population was collected in Aberdeenshire, and the Scottish definition of a heatwave is 3 days at 25°C or above (Ready Scotland 2022). However, Pilakouta et al. (2023), using a similar method, found an impact on brood size in a monogamy treatment when there was an analogous heatwave at the point of mating. The variation in these findings underscores the need for further research on the interaction between heatwaves and reproductive success, as well as additional work to outline the thermal fertility limits of *Nicrophorus vespilloides* . This will ensure that the temperatures chosen for further heatwave work are the most appropriate.

An increase in parental care behaviour may explain why we did not observe any impact of our treatments on brood size, mean larvae mass and total brood mass. Higher temperatures can lead to increased parental care investment (Pilakouta et al. 2023; Sidhu et al. 2024) as heatwave exposure may decrease parents' lifespan and trigger a terminal investment in reproduction, which leads to increased parental care and reproductive effort by the parents (Kight et al. 2000; Duffield et al. 2017) thereby increasing offspring fitness in stressful thermal environments (AlRashidi et al. 2010; Grew et al. 2019). Temperature increases are known to increase mating frequency (Kindle et al. 2006; Katsuki and Miyatake 2009), increasing mating duration (Katsuki and Miyatake 2009; Suzaki et al. 2018), shorten courtship periods (Jiao et al. 2009), and increase the likelihood of mating (Taylor et al. 2016). Potential behavioural changes in parental care, although they may themselves incur additional costs for the parents, and mating behaviour may therefore explain why we did not observe any differences in reproductive success measurements apart from the likelihood of having a brood, as they may compensate for any fertility loss during a heatwave and level out the impacts between differing levels of intrasexual and intersexual selection across our treatments.

Heatwaves negatively affected female and male survival, in line with previous studies on: butterflies ( *Pieris napi* ) (Bauerfeind and Fischer 2014), yellow dung fly ( *Scathophaga stercoraria* ) (Blanckenhorn et al. 2014), fruit flies (*Drosophila subobscura*) (Porcelli et al. 2017), bed bugs ( *Cimex lectularius* ) (Rukke et al. 2018), parasitic wasps (*Anisopteromalus calandra*) (Chevrier et al. 2019), flour beetles ( *Tribolium castaneum* ) (Sales et al. 2021) and tsetse flies (*Glossina pallidipes*) (Weaving et al. 2024). Though we did not see differences between our polyandry and monogamy treatments, which previous studies have reported (Martin and Hosken 2003; Grazer and Martin 2012; Castrezana et al. 2017). A potential explanation for this lack of effect from the polyandry treatments may be that there is less harassment from males in a heatwave, though this is unlikely, as male courtship does not seem to decrease with increased temperature (Dougherty 2021; Grandela et al. 2023; Mak et al. 2023). We also did not observe any differences in male survival between our polyandry male competition treatments; this is surprising as our treatments also differ in the levels of male–male competition, which should impact male mortality (Martin and Hosken 2003; Kvarnemo and Simmons 2013). In our heatwaves treatments, this may be due to increasing temperatures leading to decreased aggression, as observed, for example, in sand field crickets ( *Gryllus firmus* ; Nguyen and Stahlschmidt 2019), although increased aggression has also been observed during heatwaves in competitive hyperparasitoid wasp species (Chen et al. 2019). As we do not have behavioural observations of the matings, we cannot support either of these hypotheses; however, this does outline potential areas of further research. Surprisingly, we also did not see an impact of male survival on reproductive success, as we would expect that male death would limit females' mating opportunities. This may indicate that females were able to mate with both males before their death, or that mating with one male was sufficient. Nevertheless, further work is needed to distinguish among these possibilities and to determine how levels of paternity are impacted by different levels of intersexual and intersexual selection.

To conclude, differing levels of intersexual and intrasexual selection may impact fitness outcomes after a heatwave at the point of mating, although changes in mating behaviour may compensate for some of these effects. To shed light on these mechanisms, further investigation of both sexes' mating behaviour and paternity shares under thermal stress is needed.

## Author Contributions

**Karendeep K. Sidhu:** conceptualization (lead), data curation (lead), formal analysis (lead), funding acquisition (supporting), investigation (lead), methodology (lead), visualization (lead), writing – original draft (lead), writing – review and editing (lead). **Paul Caplat:** data curation (equal), formal analysis (equal), methodology (equal), supervision (supporting), visualization (supporting), writing – original draft (supporting), writing – review and editing (supporting). **Greta Bocedi:** data curation (equal), formal analysis (equal), methodology (equal), supervision (equal), visualization (equal), writing – original draft (equal), writing – review and editing (equal). **Natalie Pilakouta:** conceptualization (equal), data curation (equal), formal analysis (equal), funding acquisition (lead), methodology (equal), supervision (equal), visualization (equal), writing – original draft (supporting), writing – review and editing (supporting). **Lesley Lancaster:** conceptualization (equal), data curation (equal), formal analysis (equal), methodology (equal), supervision (equal), visualization (equal), writing – original draft (equal), writing – review and editing (equal).

## Conflicts of Interest

The authors declare no conflicts of interest.

## Supporting information


**Data S1:** ece372778‐sup‐0001‐Supinfo1.csv.

**Data S2:** ece372778‐sup‐0002‐Supinfo2.csv.

**Data S3:** ece372778‐sup‐0003‐Supinfo3.pdf.

**Data S4:** ece372778‐sup‐0004‐Appendix.docx.

## Data Availability

Data and analysis code are available in the Supporting Information.
