# Supplementary material for: The Effect of Differing Levels of Intrasexual and Intersexual Selection on Survival and Reproduction Under a Heatwave
Source: Ecol Evol. 2026 Feb 1;16(2):e72778. doi: 10.1002/ece3.72778 (PMC12862239; doi:10.1002/ece3.72778)
Supplement: Supplementary file 3 — Data S3: ece372778‐sup‐0003‐Supinfo3.pdf. [file ECE3-16-e72778-s004.pdf]

Anlaysis_code_Pub

```
load(file = 'offspirng.rda')
load(file = 'mating.rda')


write.csv(mating, "offspring.csv", row.names=F)
write.csv(offspring, "mating.csv", row.names=F)


library(ggplot2)
library(MASS)
library(pscl)
library(msme)
library(ggpubr)
library(lme4)
library(glmmTMB)
library(DHARMa)
library(RCurl)
library(dfoptim)
library(optimx)
library(emmeans)
library(car)
library(afex)
library(effects)
library(ggeffects)
```

Survival models

female suvrival models

```
mating$level_comp <- factor(mating$level_comp, levels = c("mono","COMP", "NO_
COMP"))

                                                  # Female survival

mating.mono.poly <- mating[mating$comp_or_nocomp != 'NO_COMP',  ] # subset da
ta without polyandry competition treatment

#F.survival.random.mono <- glmer(cbind(female_alive,female_dead) ~ mono_poly*
hw  + female_weight_before_mating + (1|female_family) , family = binomial(lin
k = "logit"), control=glmerControl(optimizer="bobyqa",optCtrl=list(maxfun=2e5
)),  data = mating.mono.poly)
#summary(F.survival.random.mono)

#female survival without random effects
F.survival.mono <- glm(cbind(female_alive,female_dead) ~ mono_poly*hw  + fema
```

```
le_weight_before_mating, family = binomial(link = "logit"),  data = mating.mo
no.poly)

#compare models
#AIC(F.survival.random.mono, F.survival.mono)
summary(F.survival.mono)
Anova(F.survival.mono)
marginal = emmeans(F.survival.mono, ~mono_poly*hw)
plot(allEffects(F.survival.mono))
```

Male survival

Male survival datasets graphs

```
# variables needed for data visulisation


mating$male2_alive <- ifelse(mating$male_2_survival == 'Y', "1", "0")
mating$male2_dead <- ifelse(mating$male_2_survival == 'Y', "0", "1")


mating$one_male_survive <- ifelse(mating$only_one_mals_survied == 'Y', "1", "
0")
mating$both_male_died <- ifelse(mating$both_male_survied == 'Y', "1", "0")

mating$both_male_died <- ifelse(mating$male_1_survival == "N" & mating$male_2
_survival == "N", "1", "0")
mating$both_male_survived <- ifelse(mating$male_1_survival == "Y" & mating$ma
le_2_survival == "Y", "1", "0")

mating$onlyone_male_survived <- ifelse(mating$male_1_survival == "Y" & mating
$male_2_survival == "N"| mating$male_1_survival == "N" & mating$male_2_surviv
al == "Y", "1", "0")

mating$male2_alive <- as.numeric(mating$male2_alive)
mating$male2_dead <- as.numeric(mating$male2_dead)
mating$male_2_pro_width <- as.numeric(mating$male_2_pro_width)

mating <- subset(mating, GOT.OUT== "NO") # remove NAs from survival - NAs are
for males who got out remove these values

mating1 <- subset(mating, comp_or_nocomp != "NA") ## in graphs where two male
s subset out monogamy data for graphs and percentages

poly.data <- mating[mating$mono_poly == 'POLY',  ]
mono.data <- mating[mating$mono_poly == 'MONO',  ]

#Figures
```

```
female<- ggplot(mating, aes( x = comp_or_nocomp)) +
  geom_bar(aes(fill = female_survival), position = "fill") +
  facet_grid(~ hw, labeller = labeller(hw = heatwave.treat)) +
  ylab("Females survival") +
  xlab("Treatment") +
  scale_x_discrete(labels = c("Mono", "No comp", "Comp")) +
  scale_fill_manual(values = c("red", "grey"), labels =c("No", "Yes"), name =
"Survived") +
  theme(axis.text.x = element_text(angle = 45, vjust=0.5, hjust = 0.5))


male1<- ggplot(mating, aes( x = comp_or_nocomp)) +
  geom_bar(aes(fill = male_1_survival), position = "fill") +
  facet_grid(~ hw) +
  ylab("Male 1 survival") +
  xlab("Treatment") +
  scale_x_discrete(labels = c("Mono", "No comp", "Comp")) +
  scale_fill_manual(values = c("red", "grey"), labels =c("No", "Yes"), name =
"Survived") +
  theme(axis.text.x = element_text(angle = 45, vjust=0.5, hjust = 0.5))


male2<- ggplot(mating1, aes( x = comp_or_nocomp)) +
  geom_bar(aes(fill = male_2_survival), position = "fill") +
  facet_grid(~ hw) +
  ylab(" Male 2 survival") +
  xlab("Treatment") +
  scale_x_discrete(labels = c("No comp", "Comp")) +
  scale_fill_manual(values = c("red", "grey"), labels =c("No", "Yes"), name =
"Survived") +
  theme(axis.text.x = element_text(angle = 45, vjust=0.5, hjust = 0.5))


one <- ggplot(mating1, aes( x = comp_or_nocomp)) +
  geom_bar(aes(fill = only_one_mals_survied), position = "fill") +
  facet_grid(~ hw) +
  ylab("Only one male survived") +
  xlab("Treatment") +
  scale_x_discrete(labels = c("No comp", "Comp")) +
  scale_fill_manual(values = c("grey", "red"), labels =c("No", "Yes"), name =
"Survived") +
  theme(axis.text.x = element_text(angle = 45, vjust=0.5, hjust = 0.5))


both <- ggplot(mating1, aes( x = comp_or_nocomp)) +
  geom_bar(aes(fill = both_male_survied), position = "fill") +
  facet_grid(~ hw) +
  ylab("Both males survived") +
  xlab("Treatment") +
```

```
  scale_x_discrete(labels = c("No comp", "Comp")) +
  scale_fill_manual(values = c("red", "grey"), labels =c("No", "Yes"), name =
"Survived") +
  theme(axis.text.x = element_text(angle = 45, vjust=0.5, hjust = 0.5))


ggarrange(female, male1, male2, one, both, nrow = 2, ncol = 3)
```

Male survival models

```
# Male survival

# data set to analyse male survival for both males at the same time

poly.data <- mating[mating$mono_poly == 'POLY',  ]
str(poly.data)

poly.data$male_1_pro_width[poly.data$male_1_pro_width == 0] <- NA
poly.data$male_2_pro_width[poly.data$male_2_pro_width == 0] <- NA

poly.data.m1 <- poly.data
poly.data.m1$male_dead <- poly.data.m1$male1_dead
poly.data.m1$male_alive <- poly.data.m1$male1_alive
poly.data.m1$male.id <- poly.data.m1$male_1_ID
poly.data.m1$male_pro_width <- poly.data.m1$male_1_pro_width
poly.data.m1$male_family <- poly.data.m1$male_1_family

poly.data.m2 <- poly.data
poly.data.m2$male_dead <- poly.data.m2$male2_dead
poly.data.m2$male_alive <- poly.data.m2$male2_alive
poly.data.m2$male.id <- poly.data.m2$male_2_ID
poly.data.m2$male_pro_width <- poly.data.m2$male_2_pro_width
poly.data.m2$male_family <- poly.data.m2$male_2_family
poly.data.m2<- poly.data.m2[complete.cases(poly.data.m2$male.id), ] # get rid
of any NA in male ID - possible duplicates


str(poly.data.m1)
str(poly.data.m2)

survival.combined <- rbind(poly.data.m1, poly.data.m2)

                             # male survival between polyandry treatment

survival.combined.comp <- glmer(cbind(male_alive,male_dead) ~ level_comp*hw
+ (1|male_family)  +  (1|mating_ID), family = binomial(link = "logit"),  data
= survival.combined)
```

```
## Error in pwrssUpdate(pp, resp, tol = tolPwrss, GQmat = GQmat, compDev = co
mpDev, : Downdated VtV is not positive definite

summary(survival.combined.comp)

## Error in h(simpleError(msg, call)): error in evaluating the argument 'obje
ct' in selecting a method for function 'summary': object 'survival.combined.c
omp' not found

plot(allEffects(survival.combined.comp))

## Error in allEffects(survival.combined.comp): object 'survival.combined.com
p' not found

# new data set for mono

mating$male_1_pro_width[mating$male_1_pro_width == 0] <- NA
mating$male_2_pro_width[mating$male_2_pro_width == 0] <- NA

mating.data.m1 <- mating
mating.data.m1$male_dead <- mating.data.m1$male1_dead
mating.data.m1$male_alive <- mating.data.m1$male1_alive
mating.data.m1$male.id <- mating.data.m1$male_1_ID
mating.data.m1$male_pro_width <- mating.data.m1$male_1_pro_width
mating.data.m1$male_family <- mating.data.m1$male_1_family
mating.data.m1[mating.data.m1=='NA'] <- NA # make sure all NA are coded the s
ame
mating.data.m1<- mating.data.m1[complete.cases(mating.data.m1$male.id), ] # g
et rid of any NA in male ID - possible duplicates

str(mating.data.m1)

mating.data.m2 <- mating
mating.data.m2$male_dead <- mating.data.m2$male2_dead
mating.data.m2$male_alive <- mating.data.m2$male2_alive
mating.data.m2$male.id <- mating.data.m2$male_2_ID
mating.data.m2$male_family <- mating.data.m2$male_2_family
mating.data.m2$male_pro_width <- mating.data.m2$male_2_pro_width
mating.data.m2[mating.data.m2=='NA'] <- NA
mating.data.m2<- mating.data.m2[complete.cases(mating.data.m2$male.id), ] # g
et rid of any NA in male ID - possible duplicates

str(mating.data.m2)

                        # male survival between polyandry and monogamy


survival.combined.mono <- rbind(mating.data.m1, mating.data.m2)
str(survival.combined.mono)
```

```
survival.combined.monoonepoly <- survival.combined.mono[survival.combined.mon
o$level_comp != 'NO_COMP',  ]

#survival.combined.mono.random.onepoly <- glmer(cbind(male_alive,male_dead) ~
mono_poly*hw + (1|male_family) + (1|mating_ID), family = binomial(link = "log
it"),  data = survival.combined.monoonepoly)
#summary(survival.combined.mono.random.onepoly)

survival.combined.mono.random.2.onepoly <- glmer(cbind(male_alive,male_dead)
~ mono_poly*hw + (1|male_family) + (1|mating_ID), family = binomial(link = "l
ogit"),  data = survival.combined.monoonepoly)

## boundary (singular) fit: see help('isSingular')

summary(survival.combined.mono.random.2.onepoly)

AIC(survival.combined.mono.random.2.onepoly)
```

Reproductive success

Number of eggs

```
                                        #data manipulation for models

str(offspring) # check number of offspring is number

table(offspring$comp_or_no_comp, offspring$hw)

hist(offspring$no_of_eggs, breaks = 5) # overall look at eggs

offspring1 <- offspring[!is.na(offspring$no_of_eggs), ] # subset out NA for e
ggs so either 0 or >0
all.data <- merge(offspring1, mating, by= "mating_ID", all.x=TRUE )
all.data$female_weight_before_mating <- as.numeric(all.data$female_weight_bef
ore_mating)
male2.data <- all.data[all.data$mono_poly == 'POLY', ]

poly.data <- all.data[all.data$mono_poly == 'POLY',  ]
mono.data <- all.data[all.data$mono_poly == 'MONO',  ]

#figure

offspring$comp_or_no_comp<- factor(offspring$comp_or_no_comp, levels = c("NA"
,"COMP", "NO_COMP"))
offspring <- offspring[!is.na(offspring$no_of_eggs), ]

library(ggplot2)

#figure with zeros
```

```
egg <- ggplot(offspring, aes(y = no_of_eggs, x = hw, colour = comp_or_no_comp
, fill= comp_or_no_comp)) +
         geom_boxplot(alpha = 0.4, position = position_dodge(0.8) )+
  geom_jitter(alpha = 0.5, color = "black", size = 3, shape = 21, position =
position_jitterdodge(jitter.height = 0.1, jitter.width = 0.3, dodge.width = 0
.8)) +
  theme(panel.grid.major = element_blank(), panel.grid.minor = element_blank(
),
panel.background = element_blank(), axis.line = element_line(colour = "black"
)) +
  ylab("Number of Eggs") +
  xlab("") +
  scale_x_discrete(labels = c("Control", "Heatwave")) +
  scale_fill_manual(breaks = c("NA", "COMP", "NO_COMP"), values = c("darkgrey
", "blue", "purple"), labels =c("Mono", "Comp", "Low Comp"), name = "Treatmen
t") +
  scale_colour_manual(values = c("darkgrey", "blue", "purple"), labels =c("Mo
no", "Comp", "Low Comp"), name = "Treatment")

ggsave("egg.png", width=10, height=5) # save image

print(egg)

offspring.egg.no <-  offspring[(offspring$no_of_eggs > 0 ), ] # number of egg
figure with zeros removed

#figure without zeros
egg.nozero <- ggplot(offspring.egg.no, aes(y = no_of_eggs, x = hw, colour = c
omp_or_no_comp, fill= comp_or_no_comp)) +
  geom_boxplot(alpha = 0.4, position = position_dodge(0.8) )+
  geom_jitter(alpha = 0.5, color = "black", size = 3, shape = 21, position =
position_jitterdodge(jitter.height = 0.1, jitter.width = 0.3, dodge.width = 0
.8)) +
  theme(panel.grid.major = element_blank(), panel.grid.minor = element_blank(
),
panel.background = element_blank(), axis.line = element_line(colour = "black"
)) +
  ylab("Number of Eggs") +
  xlab("") +
 scale_x_discrete(labels = c("Control", "Heatwave")) +
scale_fill_manual(breaks = c("NA", "COMP", "NO_COMP"), values = c("darkgrey",
"blue", "purple"), labels =c("Mono", "Comp", "Low Comp"), name = "Treatment")
+
 scale_colour_manual(values = c("darkgrey", "blue", "purple"), labels =c("Mon
o", "Comp", "Low Comp"), name = "Treatment")

print(egg.nozero)

ggsave("nozerosegg.png", width=10, height=5) # save images
```

```r
# data set for monogamy and poly no competition comparison

all.data <- all.data[all.data$comp_or_no_comp != 'NO_COMP',  ]

                                # models for mono and low competition

#Amount of eggs analysis with random effects without optimisation
#egg.random.nb <- glmer.nb(no_of_eggs ~ hw.x*mono_poly  + mice_weight.g. + fe
male_weight_before_mating + (1|female_family.x) + (1|male_1_family),  data =
all.data)

#Amount of eggs with random effects with optimization
#egg.random.nb.opt <- glmer.nb(no_of_eggs ~ hw.x*mono_poly  + mice_weight.g.
+ female_weight_before_mating + (1|female_family.x) + (1|male_1_family), cont
rol=glmerControl(optimizer="bobyqa",optCtrl=list(maxfun=2e5)),  data = all.da
ta)

#Amount of eggs without random effects negative binomial
egg.nb <- glm.nb(no_of_eggs ~ hw.x*mono_poly  + mice_weight.g. + female_weigh
t_before_mating,  data = all.data)
plot(egg.nb)

summary(egg.nb)


#Amount of eggs without random effects
#egg.p <- glm(no_of_eggs ~ hw.x*mono_poly  + mice_weight.g. + female_weight_b
efore_mating, family=poisson,  data = all.data)
#plot(egg.p)
#summary(egg.p)

#print(AIC(egg.random.nb, egg.random.nb.opt, egg.nb, egg.p))

summary(egg.nb)
plot(allEffects(egg.nb))

                           # Number of eggs between polyandry treatments


#Amount of eggs analysis with random effects without optimisation
#egg.random.nb.comp <- glmer.nb(no_of_eggs ~ hw.x*level_comp  + mice_weight.g
. + female_weight_before_mating + (1|female_family.x) + (1|male_1_family),  d
ata = poly.data)

#Amount of eggs with random effects with optimization
#egg.random.nb.opt.comp <- glmer.nb(no_of_eggs ~ hw.x*level_comp  + mice_weig
ht.g. + female_weight_before_mating + (1|female_family.x) + (1|male_1_family)
, control=glmerControl(optimizer="bobyqa",optCtrl=list(maxfun=2e5)),  data =
poly.data)
```

```
#Amount of eggs without random effects negative binomial
egg.nb.comp <- glm.nb(no_of_eggs ~ hw.x*level_comp  + mice_weight.g. + female
_weight_before_mating,  data = poly.data)

#Amount of eggs without random effects possion
#egg.p.comp <- glm(no_of_eggs ~ hw.x*level_comp  + mice_weight.g. + female_we
ight_before_mating, family=poisson,  data = poly.data)

# model with just levels of competition
#AIC(egg.random.nb.comp, egg.random.nb.opt.comp, egg.nb.comp, egg.p.comp)
summary(egg.nb.comp)
plot(allEffects(egg.nb.comp))
```

Brood Size and likelihood to have a brood

```
                                  #data manipulation for model

hist(offspring$no_of_larvae, breaks = 15) # overall look at number of larvae,
0 is when there were eggs but no larvae, NA is no eggs and no larvae

#table(offspring$no_of_larvae, offspring$hw) # table of how many in each cate
gory


#new variable and data set creation

all.data <- merge(offspring, mating, by= "mating_ID", all.x=TRUE )
all.data$female_weight_before_mating <- as.numeric(all.data$female_weight_bef
ore_mating)
all.data <- all.data[!is.na(all.data$no_of_larvae), ] # need to get rid of NA
larvae.data <- all.data[all.data$no_of_larvae > 0, ]
all.data$larvae_yes <- ifelse(all.data$no_of_larvae > 0, 1, 0) # create new c
olumn for binomial
all.data$larvae_no<- ifelse(all.data$no_of_larvae == 0, 1, 0) # create new co
lumn for binomial
male2.data <- all.data[all.data$mono_poly == 'POLY', ]

poly.data <- all.data[all.data$mono_poly == 'POLY',  ]
mono.data <- all.data[all.data$mono_poly == 'MONO',  ]

all.data <- all.data[all.data$comp_or_no_comp != 'NO_COMP',  ]

#figures

offspring$comp_or_no_comp<- factor(offspring$comp_or_no_comp, levels = c("NA"
,"COMP", "NO_COMP"))

brood <- ggplot(offspring, aes(y = no_of_larvae, x = hw, colour = comp_or_no_
comp, fill= comp_or_no_comp)) +
```

```
  geom_boxplot(alpha = 0.4 ) +
  geom_jitter(alpha = 0.5, color = "black", size = 3, shape = 21, position =
position_jitterdodge(jitter.height = 0.1, jitter.width = 0.3, dodge.width = 0
.8)) +
  ylab("Brood size") +
  xlab("") +
  scale_x_discrete(labels = c("Conrtol", "Heatwave")) +
  scale_fill_manual(breaks = c("NA", "COMP", "NO_COMP"), values = c("darkgrey
", "blue", "purple"), labels =c("Mono", "Comp", "No Comp"), name = "Treatment
") +
  scale_colour_manual(values = c("darkgrey", "blue", "purple"), labels =c("Mo
no", "Comp", "No Comp"), name = "Treatment")

print(brood)

offspring.larvae.no <- offspring[!is.na(offspring$no_of_larvae) & offspring$n
o_of_larvae > 0,]
#offspring.larvae.no <-  offspring[(offspring$no_of_larvae > 0 ), ] # Figure
with zeros removed
#offspring.larvae.no <- offspring[na.omit(offspring$no_of_larvae), ]
offspring$comp_or_no_comp<- factor(offspring$comp_or_no_comp, levels = c("NA"
,"COMP", "NO_COMP"))
table(offspring.larvae.no$comp_or_no_comp, offspring.larvae.no$hw )

brood.no <- ggplot(offspring.larvae.no, aes(y = no_of_larvae, x = hw, colour
= comp_or_no_comp, fill= comp_or_no_comp)) +
  geom_boxplot(alpha = 0.4 ) +
  geom_jitter(alpha = 0.5, color = "black", size = 3, shape = 21, position =
position_jitterdodge(jitter.height = 0.3, jitter.width = 0.3, dodge.width = 0
.8)) +
  theme(panel.grid.major = element_blank(), panel.grid.minor = element_blank(
),
panel.background = element_blank(), axis.line = element_line(colour = "black"
)) +
  ylab("Brood size") +
  xlab("") +
  scale_x_discrete(labels = c("Control", "Heatwave")) +
  scale_fill_manual(breaks = c("NA", "COMP", "NO_COMP"), values = c("darkgrey
", "blue", "purple"), labels =c("Mono", "Comp", "Low Comp"), name = "Treatmen
t") +
  scale_colour_manual(values = c("darkgrey", "blue", "purple"), labels =c("Mo
no", "Comp", "Low Comp"), name = "Treatment")

 print(brood.no)

ggsave("brood.no.png", width=10, height=5)

                                          ##binomial
```

```r
                # likelihood of brood between mono and low comp poly

#Larvae likelihood analysis with random effects without optimisation
#larvae.random.bio <- glmer(cbind(larvae_yes,larvae_no) ~ hw.x*mono_poly  + m
ice_weight.g. + female_weight_before_mating + (1|female_family.x) + (1|male_1
_family), family = binomial(link = "logit"),  data = all.data)

#Larvae likelihood with random effects with optimization
#larvae.random.bio.opt <- glmer(cbind(larvae_yes,larvae_no) ~ hw.x*mono_poly
+ mice_weight.g. + female_weight_before_mating + (1|female_family.x) + (1|mal
e_1_family), control=glmerControl(optimizer="bobyqa",optCtrl=list(maxfun=2e5)
), family = binomial(link = "logit"),   data = all.data)

#Larvae likelihood without random effects negative binomial
larvae.bio <- glm(cbind(larvae_yes,larvae_no) ~ hw.x*mono_poly  + mice_weight
.g. + female_weight_before_mating, family = binomial(link = "logit"),   data
= all.data)

#AIC(larvae.random.bio, larvae.random.bio.opt, larvae.bio)

summary(larvae.bio)
plot(allEffects(larvae.bio))

                     # Likelihood of Brood between Poly treatments

#Amount of eggs analysis with random effects without optimisation
#biolarvae.random.nb.comp <- glmer(cbind(larvae_yes,larvae_no) ~ hw.x*level_c
omp  + mice_weight.g. + female_weight_before_mating + (1|female_family.x) + (
1|male_1_family), family = binomial(link = "logit"),   data = poly.data)

#Amount of eggs with random effects with optimization
#biolarvae.random.nb.opt.comp <- glmer(cbind(larvae_yes,larvae_no) ~ hw.x*lev
el_comp  + mice_weight.g. + female_weight_before_mating + (1|female_family.x)
+ (1|male_1_family), family = binomial(link = "logit"),  control=glmerControl
(optimizer="bobyqa",optCtrl=list(maxfun=2e5)),  data = poly.data)

#Amount of eggs without random effects negative binomial
biolarvae.nb.comp <- glm(cbind(larvae_yes,larvae_no) ~ hw.x*level_comp  + mic
e_weight.g. + female_weight_before_mating,family = binomial(link = "logit"),
data = poly.data)


#AIC(biolarvae.random.nb.comp, biolarvae.random.nb.opt.comp, biolarvae.nb.com
p)
summary(biolarvae.nb.comp)
plot(allEffects(biolarvae.nb.comp))

                              #brood figure
```

```
brood <- ggplot(offspring, aes(y = no_of_larvae, x = hw, colour = comp_or_no_
comp, fill= comp_or_no_comp)) +
  geom_boxplot(alpha = 0.4 ) +
  geom_jitter(alpha = 0.5, color = "black", size = 3, shape = 21, position =
position_jitterdodge(jitter.height = 0.1, jitter.width = 0.3, dodge.width = 0
.8)) +
  ylab("Brood size") +
  xlab("") +
  scale_x_discrete(labels = c("No Heatwave", "Heatwave")) +
  scale_fill_manual(breaks = c("NA", "COMP", "NO_COMP"), values = c("darkgrey
", "blue", "purple"), labels =c("Mono", "Comp", "No Comp"), name = "Treatment
") +
  scale_colour_manual(values = c("darkgrey", "blue", "purple"), labels =c("Mo
no", "Comp", "No Comp"), name = "Treatment")

print(brood)

geom_jitter(width = 0.3, height = 0, alpha = 0.3, dodge.width =0.9)

# data for graph
broodlik <- offspring
broodlik$larvae_yes <- ifelse(offspring$no_of_larvae > 0, 1, 0) # create new
column for binomial
broodlik$larvae_no<- ifelse(offspring$no_of_larvae == 0, 1, 0) # create new c
olumn for binomial
broodlik$larvae_yes <- as.factor(broodlik$larvae_yes) # run s factor for bar
chart
broodlik <- broodlik[!is.na(broodlik$larvae_yes), ]

  library(ggplot2)

hw_labels <- c("NO" = "Control", "YES" = "Heatwave")

  Liklihood.having.brood <- ggplot(broodlik, aes( x = comp_or_no_comp)) +
  geom_bar(aes(fill = larvae_yes), position = "fill", width =0.5, alpha =0.5)
+
  facet_grid(~ hw, labeller = labeller(hw = hw_labels)) +
  theme_bw() +
  ylab("Likelihood of having a brood") +
  xlab("Polyandry male competition") +
  scale_x_discrete(labels = c("Mono", "Comp", "Low comp")) +
  scale_fill_manual(values = c("purple", "darkgrey"), labels =c("No", "Yes"),
name = "Brood Sucessful") +
  theme(axis.text.x = element_text(vjust=0.5, hjust = 0.5)) +
   theme(axis.line = element_line(color='black'),
    plot.background = element_blank(),
    panel.grid.minor = element_blank(),
    panel.grid.major = element_blank())

  print(Liklihood.having.brood)
```

```
#ggsave("figure_4", width=10, height=5)
```

Brood size

```
                        # Brood size between mono and low comp poly


#Amount of larvae analysis with random effects without optimisation
#larvaenumb.random.nb <- glmer.nb(no_of_larvae ~ hw.x*mono_poly  + mice_weigh
t.g. + female_weight_before_mating + (1|female_family.x) + (1|male_1_family),
data = all.data)
#summary(larvaenumb.random.nb)

#Amount of larvae with random effects with optimization
larvaenumb.random.nb.opt <- glmer.nb(no_of_larvae ~ hw.x*mono_poly  + mice_we
ight.g. + female_weight_before_mating + (1|female_family.x) + (1|male_1_famil
y), control=glmerControl(optimizer="bobyqa",optCtrl=list(maxfun=2e5)),  data
= all.data)

## Warning in checkConv(attr(opt, "derivs"), opt$par, ctrl = control$checkCon
v, :
## Model failed to converge with max|grad| = 0.0098305 (tol = 0.002, componen
t 1)

## Error in f_refitNB(lastfit, theta = exp(t), control = control): pwrssUpdat
e did not converge in (maxit) iterations

#Amount of larvae without random effects negative binomial
#larvaenumb.nb <- glm.nb(no_of_larvae ~ hw.x*mono_poly  + mice_weight.g. + fe
male_weight_before_mating,  data = all.data)
#summary(larvaenumb.nb)
#plot(larvaenumb.nb)

#Amount of larvae without random effects possion
#larvaenumb.p <- glm(no_of_larvae ~ hw.x*mono_poly  + mice_weight.g. + female
_weight_before_mating, family=poisson,  data = all.data)
#summary(larvaenumb.p)

#AIC(larvaenumb.p, larvaenumb.nb, larvaenumb.random.nb.opt, larvaenumb.random
.nb)

summary(larvaenumb.random.nb.opt)

## Error in h(simpleError(msg, call)): error in evaluating the argument 'obje
ct' in selecting a method for function 'summary': object 'larvaenumb.random.n
b.opt' not found

plot(allEffects(larvaenumb.random.nb.opt))

## Error in allEffects(larvaenumb.random.nb.opt): object 'larvaenumb.random.n
b.opt' not found
```

```
                              # Brood size between poly treatments


#Amount of larvae analysis with random effects without optimisation
#larvaenumb.random.nb.comp <- glmer.nb(no_of_larvae ~ hw.x*level_comp  + mice
_weight.g. + female_weight_before_mating + (1|female_family.x) + (1|male_1_fa
mily),  data = poly.data)

#Amount of larvae with random effects with optimization
#larvaenumb.random.nb.opt.comp <- glmer.nb(no_of_larvae ~ hw.x*level_comp  +
mice_weight.g. + female_weight_before_mating + (1|female_family.x) + (1|male_
1_family), control=glmerControl(optimizer="bobyqa",optCtrl=list(maxfun=2e5)),
data = poly.data)

#Amount of larvae without random effects negative binomial
larvaenumb.nb.comp <- glm.nb(no_of_larvae ~ hw.x*level_comp  + mice_weight.g.
+ female_weight_before_mating,  data = poly.data)

#Amount of larvae without random effects possion
#larvaenumb.p.comp <- glm(no_of_larvae ~ hw.x*level_comp  + mice_weight.g. +
female_weight_before_mating, family=poisson,  data = poly.data)

# model with just levels of competition
#AIC(larvaenumb.random.nb.comp, larvaenumb.random.nb.opt.comp, larvaenumb.nb.
comp, larvaenumb.p.comp)
summary(larvaenumb.nb.comp)
plot(allEffects(larvaenumb.nb.comp))
```

Likelihood of survival until dispersal

```
#data manipulation for models
all.data <- merge(offspring, mating, by= "mating_ID", all.x=TRUE )
all.data$female_weight_before_mating <- as.numeric(all.data$female_weight_bef
ore_mating)
all.data <- all.data[!is.na(all.data$hatching_sucess), ] # need to get rid of
NA
male2.data <- all.data[all.data$mono_poly == 'POLY', ]

hist(offspring$hatching_sucess, breaks = 15) # NA is when there is no eggs, a
nd 0 is when there are eggs but no larvae


poly.data <- all.data[all.data$mono_poly == 'POLY',  ]
mono.data <- all.data[all.data$mono_poly == 'MONO',  ]

all.data <- all.data[all.data$comp_or_no_comp != 'NO_COMP',  ]

#figure

offspring$comp_or_no_comp<- factor(offspring$comp_or_no_comp, levels = c("NA"
```

```
,"COMP", "NO_COMP"))

hatching.sucess <- ggplot(offspring, aes(y = hatching_sucess, x = hw, colour
= comp_or_no_comp, fill= comp_or_no_comp)) +
  geom_boxplot(alpha = 0.4 ) +
  geom_jitter(alpha = 0.5, color = "black", size = 3, shape = 21, position =
position_jitterdodge(jitter.height = 0.0, jitter.width = 0.3, dodge.width = 0
.9)) +
  theme(panel.grid.major = element_blank(), panel.grid.minor = element_blank(
),
panel.background = element_blank(), axis.line = element_line(colour = "black"
)) +
  ylab("Likliehood of survival until dispersal") +
  xlab("") +
  scale_x_discrete(labels = c("No Heatwave", "Heatwave")) +
  scale_fill_manual(breaks = c("NA", "COMP", "NO_COMP"), values = c("darkgrey
", "blue", "purple"), labels =c("Mono", "Comp", "Low Comp"), name = "Treatmen
t") +
  scale_colour_manual(values = c("darkgrey", "blue", "purple"), labels =c("Mo
no", "Comp", "Low Comp"), name = "Treatment")

print(hatching.sucess)

ggsave("likliehood.survival.dispersal.png", width=10, height=5)

  # Likelihood of surviving until dispersal between mono and low competition
poly

#Amount of larvae analysis with random effects without optimisation
#hatching.random.bio <- glmer(hatching_sucess~ hw.x*mono_poly  + mice_weight.
g. + female_weight_before_mating + (1|female_family.x) + (1|male_1_family), f
amily = binomial(link = "logit"),  data = all.data)

#Amount of larvae with random effects with optimization
#hatching.random.bioopt <- glmer(hatching_sucess ~ hw.x*mono_poly  + mice_wei
ght.g. + female_weight_before_mating + (1|female_family.x) + (1|male_1_family
), control=glmerControl(optimizer="bobyqa",optCtrl=list(maxfun=2e5)), family
= binomial(link = "logit"),   data = all.data)

#Amount of larvae without random effects negative binomial
hatching.bio <- glm(hatching_sucess ~ hw.x*mono_poly  + mice_weight.g. + fema
le_weight_before_mating, family = binomial(link = "logit"),   data = all.data
)

## Warning in eval(family$initialize): non-integer #successes in a binomial g
lm!

#AIC(hatching.random.bio, hatching.random.bioopt, hatching.bio)
```

```
summary(hatching.bio)
plot(allEffects(hatching.bio))

                # Likelihood of surviving until dispersal between poly

#Amount of larvae analysis with random effects without optimisation
#hatching.random.nb.comp <- glmer(hatching_sucess ~ hw.x*level_comp  + mice_w
eight.g. + female_weight_before_mating + (1|female_family.x) + (1|male_1_fami
ly), family = binomial(link = "logit"),   data = poly.data)

#Amount of larvae with random effects with optimization
#hatching.random.nbopt.comp <- glmer(hatching_sucess ~ hw.x*level_comp  + mic
e_weight.g. + female_weight_before_mating + (1|female_family.x) + (1|male_1_f
amily), family = binomial(link = "logit"),  control=glmerControl(optimizer="b
obyqa",optCtrl=list(maxfun=2e5)),  data = poly.data)

#Amount of larvae without random effects negative binomial
hatching.nb.comp <- glm(hatching_sucess ~ hw.x*level_comp  + mice_weight.g. +
female_weight_before_mating,family = binomial(link = "logit"),  data = poly.d
ata)

## Warning in eval(family$initialize): non-integer #successes in a binomial g
lm!

#AIC(hatching.random.nb.comp, hatching.random.nbopt.comp, hatching.nb.comp)
summary(hatching.nb.comp)
plot(allEffects(hatching.nb.comp))
```

Brood total weight

```
                        # Total larvae weight data manipulation
library(lme4)
all.data <- merge(offspring, mating, by= "mating_ID", all.x=TRUE )
all.data$female_weight_before_mating <- as.numeric(all.data$female_weight_bef
ore_mating)
all.data <- all.data[!is.na(all.data$larvae_weight_all), ] # need to get rid
of NA
hist(all.data$larvae_weight_all)
male2.data <- all.data[all.data$mono_poly == 'POLY', ]

poly.data <- all.data[all.data$mono_poly == 'POLY',  ]
mono.data <- all.data[all.data$mono_poly == 'MONO',  ]

# subset data set for models

all.data <- all.data[all.data$comp_or_no_comp != 'NO_COMP',  ]

#figure

offspring$comp_or_no_comp<- factor(offspring$comp_or_no_comp, levels = c("NA"
,"COMP", "NO_COMP"))
```

```
#offspring1 <-  subset(offspring, mean_larval_weight <0.5) ## in graphs where two males subset out monogamy data for graphs and percentages

library(ggplot2)

total_mass <- ggplot(offspring, aes(y = larvae_weight_all, x = hw, colour = comp_or_no_comp, fill= comp_or_no_comp)) +
  geom_boxplot(alpha = 0.4, position = position_dodge(0.8) )+
  geom_jitter(alpha = 0.5, color = "black", size = 3, shape = 21, position = position_jitterdodge(jitter.height = 0.1, jitter.width = 0.3, dodge.width = 0.8)) +
  theme(panel.grid.major = element_blank(), panel.grid.minor = element_blank(),
panel.background = element_blank(), axis.line = element_line(colour = "black")) +
  ylab("Total lavral Mass (g)") +
  xlab("") +
  scale_x_discrete(labels = c("Control", "Heatwave")) +
  scale_fill_manual(breaks = c("NA", "COMP", "NO_COMP"), values = c("darkgrey", "blue", "purple"), labels =c("Mono", "Comp", "Low Comp"), name = "Treatment") +
  scale_colour_manual(values = c("darkgrey", "blue", "purple"), labels =c("Mono", "Comp", "Low Comp"), name = "Treatment")

print(total_mass)

ggsave("totalmass.png", width=10, height=5)

                 # Total brood mass between mono and low competition poly

#Brood total weight with random effects
total.weight.random <- lmer(larvae_weight_all ~ hw.x*mono_poly  + mice_weight.g. + female_weight_before_mating + (1|female_family.x) + (1| male_1_family), data = all.data)

## boundary (singular) fit: see help('isSingular')

#brood total weight without random effects
#total.weight <- lm(larvae_weight_all ~ hw.x*mono_poly  + mice_weight.g. + female_weight_before_mating,  data = all.data)
#summary(total.weight)

#AIC(total.weight.random, total.weight)

summary(total.weight.random)
plot(allEffects(total.weight.random))

            #Total brood mass between poly
```

```r
#Brood total weight with random effects
total.weight.random.comp <- lmer(larvae_weight_all ~ hw.x*level_comp  + mice_
weight.g. + female_weight_before_mating + (1|female_family.x) + (1| male_1_fa
mily),  data = poly.data)

## boundary (singular) fit: see help('isSingular')

#brood total weight without random effects
#total.weight <- lm(larvae_weight_all ~ hw.x*level_comp  + mice_weight.g. + f
emale_weight_before_mating,  data = poly.data)

#AIC(total.weight.random, total.weight)

summary(total.weight.random.comp)
plot(allEffects(total.weight.random.comp))
```

Brood avergae weight

```r
             # mean brood mass between mono and competition low poly

#Brood mean weight with random effects
mean.weight.random <- lmer(mean_larval_weight ~ hw.x*mono_poly  + mice_weight
.g. + female_weight_before_mating + (1|female_family.x) + (1| male_1_family),
data = all.data)

## boundary (singular) fit: see help('isSingular')

#brood mean weight without random effects
#mean.weight <- lm(mean_larval_weight ~ hw.x*mono_poly  + mice_weight.g. + fe
male_weight_before_mating,  data = all.data)

#AIC(mean.weight.random, mean.weight)

summary(mean.weight.random)
plot(allEffects(mean.weight.random))

                        # Mean brood mass between poly

#Brood mean weight with random effects
mean.weight.random.comp <- lmer(mean_larval_weight ~ hw.x*level_comp  + mice_
weight.g. + female_weight_before_mating + (1|female_family.x) + (1| male_1_fa
mily),  data = poly.data)

## boundary (singular) fit: see help('isSingular')

#brood mean weight without random effects
#mean.weight.comp <- lm(mean_larval_weight ~ hw.x*level_comp  + mice_weight.g
. + female_weight_before_mating,  data = poly.data)

#AIC(mean.weight.random.comp, mean.weight.comp)
```

```
summary(mean.weight.random.comp)
plot(allEffects(mean.weight.random.comp))
```
