# Supplementary material for: The Effect of Differing Levels of Intrasexual and Intersexual Selection on Survival and Reproduction Under a Heatwave
Source: Ecol Evol. 2026 Feb 1;16(2):e72778. doi: 10.1002/ece3.72778 (PMC12862239; doi:10.1002/ece3.72778)
Supplement: Supplementary file 4 — Data S4: ece372778‐sup‐0004‐Appendix.docx. [file ECE3-16-e72778-s001.docx]

## **Appendix 1**

**Table A1:** Statistical model structure. Each model used for the analyses is outlined, listing the response variable, model type, error distribution, and fixed and random effects. The two categories of models used (monogamy and polyandry, and the difference in polyandry with differing levels of male competition) are combined, though the difference in their fixed effects is reported.

| **Response Variable** | **Model type** | **Error distribution** | **Fixed effects** | **Random effects** |
| --- | --- | --- | --- | --- |
| **Survival** | | | | |
| *Male survival* | GLMM | Binomial | heatwave and monogamy or polyandry interaction [ or interaction of heatwaves and differing levels of male competition] | Male Family ID, Mating ID |
| *Female Survival* | GLMMs | Binomial | heatwave and monogamy or polyandry interaction [ or interaction of heatwaves and differing levels of male competition] | Female family ID |
| **Reproductive success** | | | | |
| *Number of eggs* | GLM | Negative Binomial | Carcass mass, Female mass before mating, interaction between heatwaves and monogamy or polyandry [ or interaction of heatwaves and differing levels of male competition] |  |
| *Likelihood of Dispersal* | GLM | Binomial | Carcass mass, Female mass before mating, interaction between heatwaves and monogamy or polyandry [ or interaction of heatwaves and differing levels of male competition] |  |
| *Likelihood of having a brood* | GLM | Binomial | Carcass mass, Female mass before mating, interaction between heatwaves and monogamy or polyandry [ or interaction of heatwaves and differing levels of male competition] |  |
| *Brood size* | GLMMs | Negative Binomial (mono or poly) | Carcass mass, Female mass before mating, interaction between heatwaves and monogamy or polyandry [ or interaction of heatwaves and differing levels of male competition] | Female family ID and Male 1 family ID |
|  | GLM | Negative Binomial (Differing levels of male competition) |  |  |
| *Brood Total Mass* | LMMs |  | Carcass mass, Female mass before mating, interaction between heatwaves and monogamy or polyandry [ or interaction of heatwaves and differing levels of male competition] | Female family ID and Male 1 family ID |
| *Brood Mean Mass* | LMMs |  | Carcass mass, Female mass before mating, interaction between heatwaves and monogamy or polyandry [ or interaction of heatwaves and differing levels of male competition] | Female Family ID and Male 1 Family ID |
| **Effect of male survival on reproductive success in the monogamy treatment** | | | | |
| *Number of eggs* | GLMM | Negative Binomial | Carcass mass, Female mass before mating, the interaction between male survival and heatwave treatment | Male Family ID and Female Family ID |
| *Likelihood of Dispersal* | GLMM | Binomial | Carcass mass, Female mass before mating, the interaction between male survival and heatwave treatment | Male Family ID and Female Family ID |
| *Likelihood of having a brood* | GLMM | Binomial | Carcass mass, Female mass before mating, the interaction between male survival and heatwave treatment | Male Family ID and Female Family ID |
| *Brood size* | GLMM | Negative Binomial | Carcass mass, Female mass before mating, the interaction between male survival and heatwave treatment | Male Family ID and Female Family ID |
| *Brood Total Mass* | LMM |  | Carcass mass, Female mass before mating, the interaction between male survival and heatwave treatment | Male Family ID and Female Family ID |
| *Brood Mean Mass* | LMM |  | Carcass mass, Female mass before mating, the interaction between male survival and heatwave treatment | Male Family ID and Female Family ID |
| **Effect of male survival on reproductive success in the polyandry treatments** | | | | |
| *Number of eggs* | GLMM | Negative Binomial | Carcass mass, Female mass before mating, the interaction between male survival and heatwave treatment | Male Family ID and Female Family ID |
| *Likelihood of Dispersal* | GLMM | Binomial | Carcass mass, Female mass before mating, the interaction between male survival and heatwave treatment | Male Family ID and Female Family ID |
| *Likelihood of having a brood* | GLMM | Binomial | Carcass mass, Female mass before mating, the interaction between male survival and heatwave treatment | Male Family ID and Female Family ID |
| *Brood size* | GLMM | Negative Binomial | Carcass mass, Female mass before mating, the interaction between male survival and heatwave treatment | Male Family ID and Female Family ID |
| *Brood Total Mass* | LMM |  | Carcass mass, Female mass before mating, the interaction between male survival and heatwave treatment | Male Family ID and Female Family ID |
| *Brood Mean Mass* | LMM |  | Carcass mass, Female mass before mating, the interaction between male survival and heatwave treatment | Male Family ID and Female Family ID |

**Table A2:** The influence of heatwaves on male and female survival. All model for female and male survival was analysed using zero-inflated models with binomial distributions. Female survival has the covariant of female mass before mating, carcass mass, and either interaction between heatwaves and monogamy and polyandry or heatwave and different levels of female choice with random effect with female family as a random effect. The male survival models had the same covariates except for female mass before mating was excluded, and male family ID was used as a random effect instead. Bold indicates a statistically significant result.

|  | **Est** | **SE** | **Z** | **P** |
| --- | --- | --- | --- | --- |
| ***Female Survival*** |  |  |  |  |
| *Monogamy and Polyandry* |  |  |  |  |
| Monogamy or polyandry | -0.58 | 0.96 | -0.61 | 0.54 |
| Heatwave | -1.90 | 0.83 | -2.28 | **0.02** |
| Female mass before mating treatment | -3.93 | 5.95 | 0.51 | 0.51 |
| Monogamy or polyandry * Heatwave | 1.19 | 1.13 | 1.05 | 0.29 |
| *Within polyandry* |  |  |  |  |
| Level of male competition | <-0.01 | 0.83 | -0.01 | 0.99 |
| Heatwave | -0.69 | 0.78 | -0.88 | 0.38 |
| Female mass before mating treatment | 7.10 | 6.56 | 1.08 | 0.28 |
| Level of male competition * Heatwave | -0.27 | 1.06 | -0.25 | 0.80 |
| ***Male Survival*** |  |  |  |  |
| *Monogamy and Polyandry* |  |  |  |  |
| Monogamy or polyandry | -0.97 | 0.83 | -1.16 | 0.24 |
| Heatwave | -2.32 | 0.82 | -2.81 | **0.004** |
| Monogamy or polyandry * Heatwave | 1.40 | 0.96 | 1.46 | 0.14 |
| *Within polyandry* |  |  |  |  |
| Level of male competition | 0.33 | 0.55 | 0.61 | 0.54 |
| Heatwave | -0.93 | 0.49 | -1.90 | 0.06 |
| Level of male competition * Heatwave | -1.27 | 0.71 | -1.79 | 0.07 |

**Table A3:** The impact of heatwaves and monogamy or polyandry on reproductive success. The number of eggs and brood size were analysed using GLMMs with negative binomial error distributions. The likelihood of dispersal and likelihood of having a brood were analysed using GLMs with binomial distributions. Lastly, total brood mass and mean brood mass were analysed using LMMs, all models had the same covariates: carcass mass, female mass before mating, and interaction between heatwaves and monogamy or polyandry; female family ID and male one family ID were included as random effects in models that incorporated them. Bold indicates a statistically significant result.

|  | **Est** | **SE** | **Z/T** | **P** |
| --- | --- | --- | --- | --- |
| ***Number of Eggs*** |  |  |  |  |
| Heatwave | -0.31 | 0.24 | -1.25 | 0.21 |
| Monogamy or Polyandry | -0.18 | 0.23 | -0.78 | 0.44 |
| Heatwave *Monogamy or Polyandry | 0.35 | 0.34 | 1.01 | 0.31 |
| Mice mass | -0.19 | 0.12 | -1.70 | 0.09 |
| Female mass before mating | 3.64 | 2.09 | 1.65 | 0.09 |
| **Likelihood of dispersal** |  |  |  |  |
| Heatwave | -0.38 | 0.74 | -0.51 | 0.61 |
| Monogamy or Polyandry | 0.11 | 0.68 | 0.17 | 0.87 |
| Heatwave *Monogamy or Polyandry | 0.37 | 1.02 | 0.37 | 0.71 |
| Mice mass | -0.04 | 0.36 | -0.11 | 0.91 |
| Female mass before mating | 7.64 | 6.57 | 1.16 | 0.24 |
| **Likelihood of having a brood** |  |  |  |  |
| Heatwave | -0.29 | 0.72 | -0.40 | 0.69 |
| Monogamy or Polyandry | 0.29 | 0.72 | 0.40 | 0.69 |
| Heatwave *Monogamy or Polyandry | 0.24 | 1.05 | 0.23 | 0.82 |
| Mice mass | 0.06 | 0.37 | 0.15 | 0.88 |
| Female mass before mating | 7.63 | 6.46 | 1.18 | 0.24 |
| **Brood Size** |  |  |  |  |
| Heatwave | -0.54 | 0.55 | -0.98 | 0.33 |
| Monogamy or Polyandry | 0.14 | 0.53 | 0.26 | 0.79 |
| Heatwave *Monogamy or Polyandry | 0.41 | 0.77 | 0.54 | 0.59 |
| Mice mass | -0.05 | 0.30 | -0.18 | 0.86 |
| Female mass before mating | 8.43 | 5.24 | 1.61 | 0.11 |
| **Total brood Mass** |  |  | **T** |  |
| Heatwave | -1.10 | 0.76 | -1.45 | 0.16 |
| Monogamy or Polyandry | -0.41 | 0.73 | -0.57 | 0.58 |
| Heatwave *Monogamy or Polyandry | 1.26 | 1.02 | 1.23 | 0.23 |
| Mice mass | -0.06 | 0.37 | -0.15 | 0.88 |
| Female mass before mating | 12.69 | 8.30 | 1.53 | 0.14 |
| **Mean larval mass** |  |  | **T** |  |
| Heatwave | <0.01 | 0.02 | 0.50 | 0.62 |
| Monogamy or Polyandry | <0.01 | 0.02 | 0.13 | 0.90 |
| Heatwave *Monogamy or Polyandry | <0.01 | 0.27 | 0.17 | 0.87 |
| Mice mass | <-0.01 | <0.01 | 0.18 | 0.86 |
| Female mass before mating | 0.07 | 0.19 | 0.40 | 0.69 |

**Table A4:** The impact of heatwaves and different amounts of male competition in the polyandry treatment on reproductive success. The number of eggs was analysed using GLMMs with negative binomial error distributions, and brood size was analysed using a GLM also with negative binomial error distributions. The likelihood of dispersal and likelihood of having a brood were analysed using GLMs with binomial distributions. Lastly, total brood mass and mean brood mass were analysed using LMMs, all models had the same covariates: carcass mass, female mass before mating, and interaction between heatwaves and different amounts of male competition in the polyandry; female family ID and male one family ID were included as random effects in models that incorporated them. Bold indicates a statistically significant result.

|  | **Est** | **SE** | **Z** | **P** |
| --- | --- | --- | --- | --- |
| ***Number of Eggs*** |  |  |  |  |
| Heatwave | 0.05 | 0.28 | 0.17 | 0.86 |
| Level of male competition | -0.16 | 0.25 | -0.62 | 0.54 |
| Heatwave *Level of male competition | 0.26 | 0.40 | 0.65 | 0.51 |
| Mice mass | -0.06 | 0.11 | -0.51 | 0.61 |
| Female mass before mating | 0.03 | 2.56 | 0.01 | 0.99 |
| **Likelihood of dispersal** |  |  |  |  |
| Heatwave | 0.03 | 0.73 | 0.04 | 0.97 |
| Level of male competition | 0.80 | 0.71 | 1.13 | 0.26 |
| Heatwave *Level of male competition | -0.72 | 1.06 | -0.68 | 0.49 |
| Mice mass | 0.28 | 0.29 | 0.95 | 0.34 |
| Female mass before mating | 17.20 | 7.47 | 2.30 | **0.02** |
| **Likelihood of having a brood** |  |  |  |  |
| Heatwave | -0.01 | 0.82 | -0.02 | 0.99 |
| Level of male competition | 2.57 | 1.27 | 2.02 | **0.04** |
| Heatwave *Level of male competition | -3.14 | 1.56 | -2.01 | **0.04** |
| Mice mass | 21.07 | 9.24 | 2.28 | **0.02** |
| Female mass before mating | 0.03 | 2.56 | 0.01 | 0.99 |
| **Brood Size** |  |  |  |  |
| Heatwave | -0.12 | 0.44 | -0.28 | 0.78 |
| Level of male competition | 0.03 | 0.41 | 0.08 | 0.93 |
| Heatwave *Level of male competition | 0.02 | 0.63 | 0.03 | 0.98 |
| Mice mass | 0.07 | 0.17 | 0.38 | 0.70 |
| Female mass before mating | 6.68 | 3.89 | 1.71 | 0.08 |
| **Total brood Mass** |  |  |  |  |
| Heatwave | <0.01 | 0.78 | <0.01 | 0.99 |
| Level of male competition | -0.57 | 0.68 | -0.85 | 0.40 |
| Heatwave *Level of male competition | 1.50 | 1.12 | 1.34 | 0.19 |
| Mice mass | -0.44 | 0.36 | -1.26 | 0.21 |
| Female mass before mating | 6.61 | 7.35 | 0.90 | 0.38 |
| **Mean larval mass** |  |  |  |  |
| Heatwave | 0.01 | 0.02 | 0.72 | 0.48 |
| Level of male competition | <0.01 | -0.01 | 0.25 | 0.81 |
| Heatwave *Level of male competition | <-0.01 | 0.02 | -0.19 | 0.85 |
| Mice mass | <-0.01 | <0.01 | -1.27 | 0.21 |
| Female mass before mating | 0.08 | 0.15 | 0.55 | 0.59 |

**Table A5:** The impact of male survival and heatwaves on reproductive success in the monogamy treatment. The number of eggs and brood size was analysed using GLMMs with negative binomial error distributions, The likelihood of dispersal and probability of having a brood were analysed using GLMMs with binomial distributions. Lastly, total brood mass and mean brood mass were analysed using LMMs; all models had the same covariates: carcass mass, female mass before mating, and interaction between heatwaves and male survival, and the same random effects female family ID and male family ID. Bold indicates a statistically significant result.

| **Impact of male survival on monogamy treatment reproductive success** | | | | |
| --- | --- | --- | --- | --- |
|  | **Est** | **SE** | **Z/T** | **P** |
| ***Number of Eggs*** |  |  | **Z** |  |
| Heatwave | -0.31 | 0.18 | -1.74 | 0.08 |
| Mice mass | -0.17 | 0.07 | -2.33 | **0.02** |
| Male Survival | -0.04 | 0.16 | -0.29 | 0.77 |
| Female mass before mating | 5.32 | 1.18 | 4.51 | **<0.01** |
| Heatwave * Male survival | 0.16 | 0.22 | 0.71 | 0.48 |
| **Likelihood of dispersal** |  |  |  |  |
| Heatwave | -0.09 | 1.65 | -0.06 | 0.96 |
| Mice mass | 0.46 | 0.56 | 0.82 | 0.41 |
| Male Survival | 0.94 | 1.50 | 0.62 | 0.54 |
| Female mass before mating | 3.86 | 9.11 | 0.42 | 0.68 |
| Heatwave * Male Survival | -0.13 | 1.90 | -0.07 | 0.94 |
| **Likelihood of having a brood** |  |  |  |  |
| Heatwave | 0.43 | 1.44 | 0.30 | 0.76 |
| Mice mass | 0.24 | 0.54 | 0.45 | 0.66 |
| Male Survival | 1.38 | 1.36 | 1.01 | 0.31 |
| Female mass before mating | 3.56 | 8.78 | 0.41 | 0.69 |
| Heatwave * Male Survival | -0.57 | 1.75 | -0.32 | 0.75 |
| **Brood Size** |  |  |  |  |
| Heatwave | 1.23 | 1.76 | 0.70 | 0.49 |
| Mice mass | 0.53 | 0.48 | 1.11 | 0.27 |
| Male Survival | 2.59 | 1.75 | 1.48 | 0.14 |
| Female mass before mating | 5.41 | 7.72 | 0.70 | 0.49 |
| Heatwave * Male Survival | -2.58 | 2.12 | -1.22 | 0.22 |
| **Total brood Mass** |  |  | **T** |  |
| Heatwave | -1.26 | 1.52 | 0.83 | 0.42 |
| Mice mass | 0.42 | 0.56 | 0.76 | 0.46 |
| Male Survival | -0.09 | 1.44 | -0.07 | 0.95 |
| Female mass before mating | 12.04 | 09.58 | 1.26 | 0.24 |
| Heatwave * Male Survival | 0.85 | 1.69 | 0.50 | 0.62 |
| **Mean larval mass** |  |  | **T** |  |
| Heatwave | <0.01 | 0.02 | 0.22 | 0.83 |
| Mice mass | -0.01 | <0.01 | -1.77 | **0.08** |
| Male Survival | -0.02 | 0.02 | -1.02 | 0.32 |
| Female mass before mating | 0.02 | 0.14 | 0.17 | 0.86 |
| Heatwave * Male Survival | -0.01 | 0.03 | -0.23 | 0.67 |
|  |  |  |  |  |

**Table A6:** The impact of heatwaves and different amounts of male competition in the polyandry treatment on reproductive success. The number of eggs was analysed using GLMMs with negative binomial error distributions, and brood size was analysed using a GLM also with negative binomial error distributions. The likelihood of dispersal and likelihood of having a brood were analysed using GLMs with binomial distributions. Lastly, total brood mass and mean brood mass were analysed using LMMs, all models had the same covariates: carcass mass, female mass before mating, and interaction between heatwaves and different amounts of male competition in the polyandry; female family ID and male one family ID were included as random effects in models that incorporated them. Bold indicates a statistically significant result.

| **Impact of male survival on polyandry treatments reproductive success** | | | | |
| --- | --- | --- | --- | --- |
|  | Est | SE | Z/T value | Pr |
| ***Number of Eggs*** |  |  | Z |  |
| Heatwave | 0.021 | 0.29 | 0.07 | 0.94 |
| Competition | -0.14 | 0.25 | -0.57 | 0.57 |
| Mice mass | -0.07 | 0.11 | -0.65 | 0.52 |
| At least one male survived | 0.65 | 0.37 | 1.77 | 0.08 |
| All males survived | 0.57 | 0.41 | 1.39 | 0.16 |
| Female Mass before mating | 0.22 | 2.55 | 0.09 | 0.93 |
| Heatwave * Competition | 0.58 | 0.42 | 1.36 | 0.17 |
| ***Likelihood of survival to dispersal*** |  |  | Z |  |
| Heatwave | 0.18 | 0.77 | 0.23 | 0.82 |
| Competition | 0.78 | 0.71 | 1.10 | 0.27 |
| Mice mass | 0.28 | 0.30 | 0.95 | 0.34 |
| At least one male survived | 0.20 | 1.16 | 0.17 | 0.86 |
| All males survived | 0.59 | 1.33 | 0.45 | 0.66 |
| Female Mass before mating | 17.90 | 7.52 | 2.38 | **0.02** |
| Heatwave * Competition | -0.51 | 1.22 | -0.42 | 0.67 |
| ***Likelihood of having a brood*** |  |  | Z |  |
| Heatwave | 0.04 | 1.61 | 0.03 | 0.98 |
| Competition | 2.59 | 2.78 | 0.93 | 0.35 |
| Mice mass | 0.34 | 0.66 | 0.53 | 0.60 |
| At least one male survived | 0.32 | 2.26 | 0.14 | 0.89 |
| All males survived | 0.78 | 2.75 | 0.28 | 0.77 |
| Female Mass before mating | -2.55 | 3.46 | -0.74 | 0.46 |
| **Brood size** |  |  | Z |  |
| Heatwave | -0.12 | 0.45 | -0.26 | 0.79 |
| Competition | 0.03 | 0.41 | 0.07 | 0.94 |
| Mice mass | 0.10 | 0.17 | 0.59 | 0.55 |
| At least one male survived | 0.53 | 0.70 | 0.77 | 0.44 |
| All males survived | 0.53 | 0.79 | 0.67 | 0.50 |
| Female Mass before mating | 7.01 | 3.95 | 1.78 | 0.08 |
| Heatwave * Competition | 0.24 | 0.73 | 0.33 | 0.74 |
| ***Total brood mass*** |  |  | T |  |
| Heatwave | -0.16 | 0.82 | -0.19 | 0.85 |
| Competition | -0.54 | 0.69 | -0.79 | 0.44 |
| Mice mass | -0.44 | 0.37 | -1.20 | 0.24 |
| At least one male survived | 6.44 | 7.57 | 0.85 | 0.40 |
| All males survived | 0.25 | 1.44 | 0.17 | 0.87 |
| Female Mass before mating | -0.28 | 1.59 | -0.18 | 0.86 |
| Heatwave * Competition | 1.41 | 1.33 | 1.05 | 0.30 |
| ***Mean brood mass*** |  |  | T |  |
| Heatwave | <0.01 | 0.02 | 0.38 | 0.70 |
| Competition | <-0.01 | 0.01 | 0.31 | 0.76 |
| Mice mass | -0.01 | <0.01 | -1.50 | 0.14 |
| At least one male survived | 0.04 | 0.15 | 0.30 | 0.76 |
| All males survived | -0.02 | 0.03 | -0.74 | 0.46 |
| Female Mass before mating | -0.04 | 0.03 | -1.19 | 0.24 |
| Heatwave * Competition | -0.02 | 0.03 | -0.75 | 0.46 |
